# IRF4b and IRF8 Negatively Regulate RLR-Mediated NF-κB Signaling by Targeting MITA for Degradation in Teleost Fish

**DOI:** 10.3389/fimmu.2022.858179

**Published:** 2022-03-03

**Authors:** Xiaolong Yan, Xueyan Zhao, Ming Zhou, Yuena Sun, Tianjun Xu

**Affiliations:** ^1^ Laboratory of Fish Molecular Immunology, College of Fisheries and Life Science, Shanghai Ocean University, Shanghai, China; ^2^ National Pathogen Collection Center for Aquatic Animals, Shanghai Ocean University, Shanghai, China; ^3^ Key Laboratory of Exploration and Utilization of Aquatic Genetic Resources, Shanghai Ocean University, Ministry of Education, Shanghai, China; ^4^ Laboratory of Marine Biology and Biotechnology, Qingdao National Laboratory for Marine Science and Technology, Qingdao, China

**Keywords:** MITA, IRF4b, IRF8, NF-κB, ubiquitination

## Abstract

Mediator of IRF3 activation (MITA) is a significant signal adaptor in the retinoic acid-inducible gene-I like receptor (RLR) signaling pathway and plays an important role in the innate immune system. As a transcription factor, nuclear factor kappa B (NF-κB) can be available in many signaling pathways including the RLR signaling pathway and relative to biological processes like immune responses. In this study, it is determined that IRF4b and IRF8 can have a negative effect on NF-κB signaling pathway mediated by MITA in fish. Firstly, it is found that IRF4b and IRF8 have an inhibitory function on MITA-mediated NF-κB signaling pathway. It is interesting that IRF4b and IRF8 have similar functions to achieve precise downregulated and the degradation of MITA through the ubiquitin-proteasome pathway. IRF is taken as the core domain of IRF4b or IRF8 for the downregulation to MITA. This study provides data on MITA-mediated NF-κB signaling pathway in teleost fish and provides new insights into the regulatory mechanism in fish immune system.

## Introduction

Pathogens produce a series of conserved components, which are called pathogen-associated molecular patterns (PAMPs) in the process of invading the host cells. As pattern recognition receptors (PRRs) in the host cells can recognize PAMPs, a series of signaling cascades are induced (1). Downstream signaling pathways are activated, and the productions of type I interferon, proinflammatory cytokines, and chemokines are induced to defend against invading pathogens (2). These PRRs mainly consist of four types, which include Toll-like receptors (TLRs), retinoic acid-inducible gene-I-like receptors (RLRs), C-type lectin receptors (CLRs), and nucleotide oligomerization domain (NOD)-like receptors (NLRs) (3–5). Among the four types of PPRs, TLRs and RLRs are relatively typical as they are highly conserved in vertebrates and play an important role in the innate immune system. After being recognized by corresponding ligands, TLRs can cause the activation of two downstream signaling pathways. One is MyD88-dependent pathway, and the other is MyD88-independent pathway, which is also called the TRIF-dependent pathway (6, 7). All the members of TLRs contain a TIR domain, and MyD88 also has a TIR domain. As an important molecule of TLR signaling pathway, MyD88 first binds to TLRs through the TIR domain. The complex then combines with interleukin-1 receptor-associated kinase 4 (IRAK-4), which results in the activation of interleukin-1 receptor-associated kinase 1 (IRAK-1) and tumor necrosis factor receptor-associated factor 6 (TRAF6). Nuclear factor κB (NF-κB) is eventually activated, which can promote the expression of some inflammatory factors by a range of reactions (8–10).

Most studies focus on TLR signaling pathway while RLR signaling pathway has received more and more attention. TLRs can recognize the external stimuli from bacteria, viruses, fungi, and protozoa, while RLRs can only recognize the stimulus from viruses. So far, three members of RLRs have been identified, and they are retinoic acid-induced gene I (RIG-I), melanoma differentiation-associated gene 5 (MDA5), and laboratory of genetics and physiology 2 (LGP2) (11). RIG-I and MDA5 can interact with mitochondrial antiviral signaling protein (MAVS), a CARD-containing adaptor protein, through their CARD domain (12–14). Through the activation of MAVS, the signal is transmitted to the downstream TRAF3, TBK1, and inducible IκB kinase (IKK-i), and finally IRF3 and IRF7 are phosphorylated. The phosphorylated IRF3 and IRF7 are transformed into nucleus, which can induce the production of type I interferon (IFN) (15). Mediator of IRF3 activation (MITA, also called STING) has been identified in many vertebrates, and it plays a role of a junction molecule in RLR signaling pathway, or rather, MITA is a signal adaptor that connects MAVS to the downstream molecules in RLR signaling pathway (16). MITA locates in the mitochondrial outer membrane and endoplasmic reticulum and is taken as an IFN-stimulating factor that is widely expressed in a variety of tissues and cells. As a result, MITA may play an extremely important role in immune regulation (17).

In the antiviral immune response, the host cells recognize the invading viruses through RLRs and trigger the downstream signal pathway, which eventually cause the host cells to produce IFN or other cytokines to defend against the viruses. While the host cells resist the invasion of the viruses, it is necessary to maintain the immune balance, in which the regulation of RLR signaling pathway is particularly critical. The genes that can participate in the regulation of RLR signaling pathway are mainly divided into two types: noncoding genes and coding genes. Studies have found that microRNAs are typical noncoding genes. MiR-4661 and miR-378 can negatively regulate the expression of IFN-α directly, thus inhibiting the antiviral innate immune response. It is reported that MITA has an effect on the production of IFN-β (18, 19). Interestingly, it is found that miR-24 can regulate the expression of MITA after transcription (20). Although there are a few studies on the regulation of target molecules by noncoding genes, more and more attentions are put into the studies on the mechanism of regulating signaling pathways through the interactions between junction molecules and target proteins, especially in mammals. A previous study shows that NLRX1 plays a negative regulatory role in MAVS-mediated antiviral response which is mediated by inhibiting the interactions between virus-induced RIG-like helicase (RLH) and MAVS (21). As a physiological inhibitor of MDA5, DAK can specifically inhibit the innate antiviral signal transduction which was mediated by MDA5 (22). In addition, antiviral signals related to RLRs can be activated when MAVS recruits TRAF6 (23). In a word, regulating the junction molecules and the target signal proteins in RLR signaling pathway is an important part of the immunomodulatory mechanism.

Many studies that focus on the functions of MITA in mammals are available, but there are only a few when it comes to fish. In this study, we focus on the research regarding the influence of IRF4b and IRF8 on MITA in teleost fish. The existence of either IRF4b or IRF8 can inhibit the activation level of MITA-mediated NF-κB signaling pathway. When IRF4b or IRF8 is overexpressed, the protein level of MITA drops in an obvious way while the knockdown of IRF4b or IRF8 presents the opposite results. In addition, it is confirmed that both IRF4b and IRF8 promote the degradations of MITA through ubiquitin-proteasome pathway. This study not only provided evidence for the mechanism of IRFs regulating MITA in fish but also enriched the content of RLR signaling pathway in fish. What is more, it provides a new sight for the regulatory mechanism in vertebrates.

## Materials and Methods

### Sample and Challenge

The healthy juvenile fish of miiuy croaker (Miichthys Miiuy) with a body weight of 25~30 g were reared in 25°C inflatable seawater tanks for at least 1 week, and pathogen infection experiments were then carried out. Healthy fish were randomly divided into the control group and the injection group. In the injection group, fish were further divided into several groups and kept in different tanks according to the different stimulus. The stimulus were the suspension of poly(I:C) (5 mg/ml, *In vivo*Gen) and Siniperca chuatsi rhabdovirus (SCRV), and the fish were injected with them in a dose of 0.1 ml, respectively. The fish in the control group were injected with 0.1 ml normal saline as a control. The fish in the three groups were all killed for liver at different time points (0, 6, 12, 24, 36, 48, and 72 h) after injection, and at least three samples in each group were collected at each time point. All animal experiments were conducted in accordance with the guidelines for the Care and Use of Experimental Animals issued by the National Institutes of Health, and the experiments were approved by the Research Ethics Committee of Shanghai Ocean University (No. SHOU-DW-2018-047).

### Plasmid Construction

The open reading frame (ORF) of miiuy croaker MITA gene was cloned from the cDNA of miiuy croaker into the *Hind* III and *EcoR* I sites of pcDNA3.1 with a Myc tag. The ORF of MITA was cloned into the same restriction enzyme sites as pcDNA3.1 with a Myc tag in pEGFP-N1 with a green fluorescent protein (GFP) tag. The ORF of IRF4b gene was cloned in cDNA of miiuy croaker into the *Hind* III and *EcoR* I sites of pcDNA3.1 with a Flag tag. The ORF of IRF8 gene was cloned from the cDNA of miiuy croaker into *BamH* I and *Xba* I sites of pcDNA3.1 with a Flag tag. Based on the recombinant plasmid of IRF4b, the relative mutations of IRF4b, including IRF4bΔIRF and IRF4bΔIRF3, were generated by specific primers through PCR. The mutations of IRF8, which included IRF8ΔIRF and IRF8ΔIRF3, were generated in the similar way as the mutations of IRF4b. The IRF4b-shRNA was designed and ligated into *BamH* I and *EcoR* I of pSIREN-RetroQZsGreen1 vector and so was the IRF8-shRNA. The pRK5-HA-ubiquitin-WT (ubiquitin-HA) plasmid was purchased from Addgene (Watertown, MA, USA). All recombinant plasmids were affirmed by Sanger sequencing. All of the plasmids were then extracted using Endotoxin Free Plasmid DNA Miniprep Kit (Tiangen, Beijing, China). Primer sequences are listed in **Supplemental Table 1**.

### Cell Culture and Transient Transfections

Epithelioma papulosum cyprini (EPC) cells were cultured in medium 199 (Hyclone, Logan, UT, USA), which contains 10% fetal bovine serum (FBS, Gibco, Waltham, MA, USA), 2 mM l-glutamine, 100 U/ml penicillin, and 100 mg/ml streptomycin, in a 26°C incubator with 5% CO_2_. The cell line of miiuy croaker kidney (MKC) was cultured in L-15 medium supplemented with 20% FBS at 26°C (24). HEK293 cells were cultured in DMEM medium in a humid environment containing 5% CO_2_ at 37°C, and 10% FBS, 2 mM l-glutamine, 100 U/ml penicillin, and 100 mg/ml streptomycin were contained in the DMEM medium. The plasmids were transfected in cells by Lipofectamine 2000™ (Invitrogen, Waltham, MA, USA). Furthermore, the proteasome inhibitor (MG132, CAS number: 1211877-36-9, Sigma, St. Louis, MO, USA) or cycloheximide (CHX, CAS number: 66-81-9, Beyotime) was added into medium at 24 h after transfection, and the final concentrations were 30 μM/ml and 100 μg/ml, respectively (25).

### Luciferase Reporter Assays

The expression plasmids and reporter gene plasmids like NF-κB, IL-1β, and IL-8 were transfected in EPC cells, and Renilla luciferase reporter plasmid (pRL-TK) was regarded as the internal control. The ratio between pRL-TK and reporter gene plasmids was 1:10. The control group was added with the same amount of empty vector as the experimental group to keep the same amount of total transfection in the whole group of experiments. The luciferase activity was measured using Dual-Luciferase Reporter Assay System (Promega, Madison, WI, USA). In order to obtain results, each experiment was carried out at least three times independently (26).

### Immunoblot Assays

The cells were washed for three times by sterile and cold PBS, then the cells were lysed by Western and IP cell lysis buffer (Beyotime, 20 mM Tris (pH 7.5), 150 mM NaCl, 1% Triton X-100). The concentrations of the proteins were measured through BCA assay (Pierce). The same amount of protein samples were mixed with 2× SDS loading buffer, and they were loaded into SDS-PAGE. Through Bio-Rad Trans Blot Turbo System, the proteins were transferred to PVDF membrane (Millipore, Burlington, MA, USA) by the semidry process. The membranes were blocked in 5% skimmed milk solution at room temperature for 90 min and incubated with suitable primary antibodies at 4°C overnight. The primary antibodies used in this study were against Myc, Flag, HA, GFP Tag (Santa Cruz, Santa Cruz, CA, USA), GAPDH, and tubulin. Using the TBST buffer, the membranes were washed for three times, and they were then incubated with the secondary antibody at room temperature on the rocker platform for 60 min. Finally, proteins were detected by WesternBright™ ECL (Advansta, San Jose, CA, USA), and cold CCD camera was used for digital imaging.

### Immunoprecipitation Assays

For immunoprecipitation (IP) experiments, HEK293 cells were seeded into 10 cm^2^ plate overnight and transfected with a total 5 µg plasmids. After 36 h transfection, the cells were washed for three times with ice-cold PBS, and the cells were lysed with 500 µl Western and IP lysis buffer, which contain protease inhibitor cocktail (Bitake), at 4°C for 30 min on a rocker platform. The cell samples were centrifuged at 14,000×*g* for 15 min at 4°C. After centrifugation, the supernatants were transferred to new centrifuge tubes and gently shaken overnight at 4°C with 50 µl protein A+G (Sigma) and 1 µg anti-Myc monoclonal antibody (Sigma). In the next day, the beads were collected after centrifuging at 2,500×*g* for 5 min at 4°C. After being washed with Western and IP lysis buffer for 5 times, the beads were finally mixed with 60 µl 2× SDS loading buffer. The immunoprecipitates and the whole cell lysates were analyzed by immunoblotting.

### Fluorescent Microscopy

HEK293 cells were cultivated onto 24-well plates and transfected using Lipofectamine 2000™ (Invitrogen) with corresponding plasmids for 48 h, and the images were obtained by a fluorescence microscope (Leica, Wetzlar, Germany).

### Statistical Analysis

All the experiments were performed independently at least three times (*n* ≥ 3). The relative data of the expression of genes were obtained by 2^−ΔΔCT^ method, and the comparisons between groups were analyzed by one-way analysis of variance (ANOVA) followed by Duncan’s multiple comparison tests. Results were expressed as mean ± SE (standard error), and the *p* values <0.05 was considered to be statistically significant (27).

## Results

### IRF4b and IRF8 Are Upregulated After Induced by Poly(I:C) and SCRV

**Figure 1:**
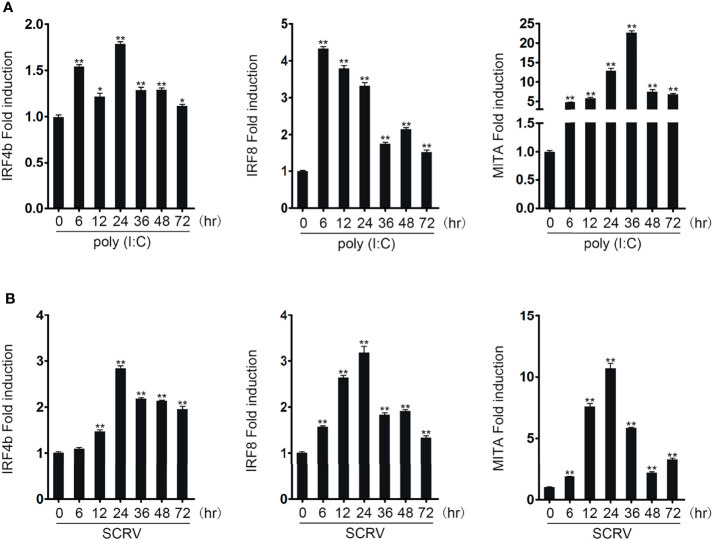
IRF4b and IRF8 are upregulated after poly(I:C) and SCRV induction. The expression patterns of IRF4b, IRF8, and MITA were analyzed using the liver samples of fish obtained at 0, 6, 12, 24, 36, 48, and 72 h after being injected with poly(I:C) **(A)** and SCRV **(B)** by qRT-PCR, respectively. ^*^ *p* < 0.05; ^**^ *p* < 0.01. All the experiments were performed independently at least three times.

To know whether the expressions of IRF4b, IRF8, and MITA could be affected by pathogen stimulation, miiuy croaker were stimulated with poly(I:C) and SCRV, and the expressions in liver was detected by qRT-PCR (**Figures 1A, B**). When stimulated with poly(I:C), compared with the expressions of the three genes at 0 h as the control, the expressions of the three genes all increased. With the extension of stimulation time, the expressions of these three genes also showed different changes, and the maximum expression of three different genes appeared at different times. So did when stimulated with SCRV. It was vividly shown that no matter what kind of stimulation was used, the maximum expression of MITA always appeared later than that of IRF4b and IRF8. Therefore, it could be inferred that IRF4b and IRF8 may have an effect on the MITA under the stimulation of poly(I:C) or SCRV.

### IRF4b and IRF8 Negatively Influence the MITA-Mediated NF-κB Signaling Pathway

**Figure 2:**
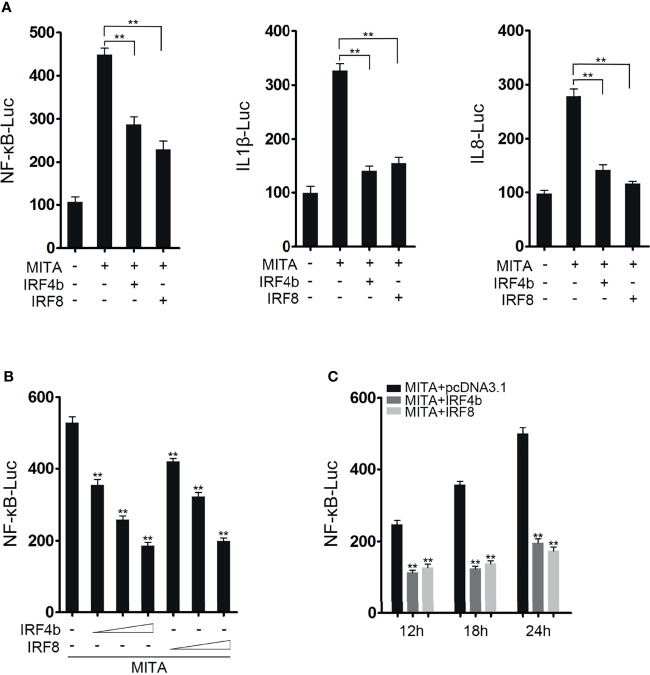
IRF4b and IRF8 negatively influence the MITA-mediated NF-κB signaling pathway. **(A)** EPC cells were seeded onto 24-well plates overnight and cotransfected with MITA, pcDNA3.1, IRF4b, IRF8, and the reporter genes such as NF-κB (left), IL-1β (middle), and IL-8 (right), respectively. The dose of each plasmid was 0.2 μg (0.25 μg for every kind of reporter gene). Luciferase activity was measured at 24 h after transfection. **(B)** The concentration gradient experiments of IRF4b and IRF8 were performed in the following doses: 0.05, 0.1, or 0.2 μg, and MITA and NF-κB were also transfected into EPC cells. The cells were lysed at 24 h for luciferase reporter assays. **(C)** MITA, IRF4b, IRF8, and the reporter gene of NF-κB were transfected into EPC cells, and the cells were randomly divided into three groups according to the different time points. After being lysed at 12, 18, and 24 h, luciferase reporter assays were performed. ^**^ *p* < 0.01. All the experiments were performed independent at least three times.

It was known that MITA could mediate NF-κB signaling pathway in previous study. In order to confirm the conjecture above, the influences of IRF4b and IRF8 on MITA-mediated NF-κB signaling pathway were studied. As shown in **Figure 2A**, MITA did activate the promoter activity of NF-κB, and the promoter activity of NF-κB decreased with the presence of IRF4b or IRF8 when compared with the results that only MITA existed. Similar results are received when they are related to the promoter of IL-1β or IL-8. To confirm the influence of IRF4b or IRF8 on MITA, concentration gradient experiments of IRF4b and IRF8 were conducted, as shown in **Figure 2B**. With the gradual increased doses of IRF4b and IRF8, the activation level of NF-κB also decreased gradually. IRF4b or IRF8 was then transfected with MITA into EPC cells, as designed in **Figure 2C**, and luciferase activity was checked at 12, 18, and 24 h, respectively. The results demonstrated that both the existence of IRF4b and IRF8 could have an inhibitory effect on the activation of NF-κB which was mediated by MITA. In other words, the effects of IRF4b or IRF8 on MITA-mediated signaling pathway may be caused by some interaction between IRF4b or IRF8 and MITA.

### IRF4b and IRF8 Promote MITA Degradation

**Figure 3:**
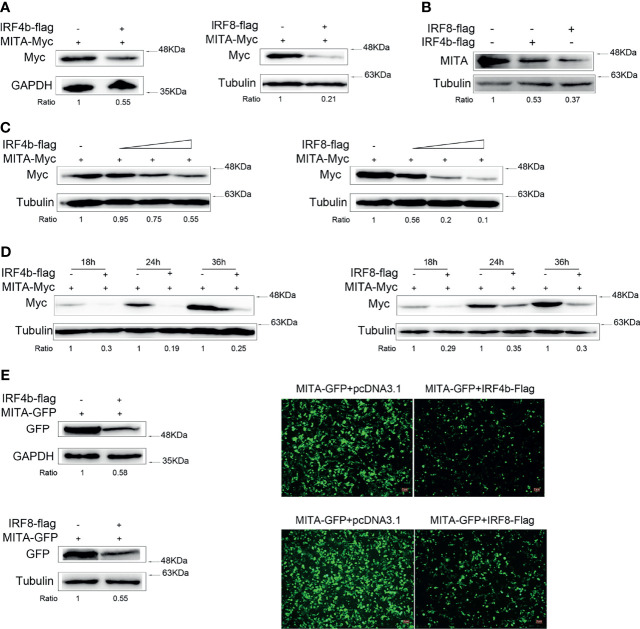
IRF4b and IRF8 promote MITA degradation. **(A)** EPC cells were seeded onto 12-well plates to transfect with pcDNA3.1, IRF4b-Flag, or IRF8-Flag together MITA-Myc in a 1 μg dose for each plasmid. At 24 h after transfection, the protein level of MITA was examined by immunoblot assays and normalized to GAPDH and tubulin. **(B)** A total of 0.8 μg of pcDNA3.1, IRF4b-Flag, and IRF8-Flag was transfected into MKC, respectively. The cells were then lysed 24 h later, and the expression of endogenous MITA was checked by immunoblot assays and normalized to tubulin. **(C)** The concentration experiments of IRF4b and IRF8 were carried out. Together with the increasing doses (0.2, 0.4, or 0.8 μg) of IRF4b or IRF8, 0.8 μg of MITA was transfected into EPC cells, in which the pcDNA3.1 was used to make the balance of the dose, and MITA was examined by immunoblot assays finally. **(D)** EPC cells were transfected with IRF4b or IRF8 together with MITA, and cells were lysed at different time points to check the expression of MITA by immunoblot assays and be normalized to tubulin. **(E)** HEK293 cells were seeded onto 12-well plates overnight and transfected with MITA-GFP, IRF4b-Flag, or IRF8-Flag. Green signals arising from MITA-GFP was detected by fluorescence microscopy (right), and the cells were lysed for immunoblot assays (left) the fluorescence intensity was observed by Leica DMiL8 fluorescence microscope. Scale bar, 20 mm; original magnification ×10. All the experiments were performed independently at least for three times.

In order to find out the relationship between IRF4b or IRF8 and MITA, we mainly studied the changes of the expressions of MITA. Expression plasmids for MITA were transfected with IRF4b or IRF8 into EPC cells for immunoblot assays (**Figure 3A**). The protein level of MITA decreased obviously with the participation of IRF4b or IRF8. To explore the influence of IRF4b or IRF8 on the expression of endogenous MITA, the expression plasmids of IRF4b and IRF8 were transfected into MKC, respectively, and the expression of endogenous MITA was examined by immunoblot assays (**Figure 3B**). Similar results were obtained as in **Figure 3A**. To further confirm the inhibitory effects of IRF4b or IRF8 on the expression of MITA, concentration gradient experiments and different time points experiments were conducted, as shown in **Figures 3C, D**. The results demonstrated that both IRF4b and IRF8 could inhibit the expression of MITA. MITA-GFP was transfected with IRF4b or IRF8 into HEK293 cells (**Figure 3E**). As shown in the pictures, the green signals of MITA were weaker with the presence of IRF4b or IRF8 when compared with the control, and the results of immunoblot assays also presented the similar trends. As a result, IRF4b and IRF8 could promote the degradation of MITA.

### Effects of the Knockdown of IRF4b or IRF8 on MITA

**Figure 4:**
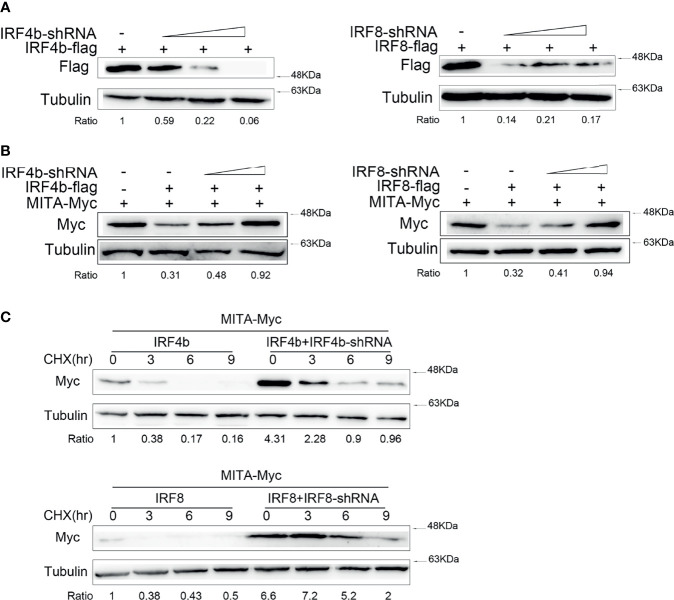
Effects of the knockdown of IRF4b or IRF8 on MITA. **(A)** The validity of IRF4b-shRNA or IRF8-shRNA was checked after transfecting IRF4b and IRF4b-shRNA or IRF8 and IRF8-shRNA into EPC cells, and the expression of IRF4b or IRF8 was examined by immunoblot assays at 24 h after transfection. **(B)** MITA and IRF4b were cotransfected with IRF4b-shRNA (0.2 and 0.4 μg) into EPC cells, and MITA, IRF8, and IRF8-shRNA were transfected in the same way as MITA, IRF4b, and IRF4b-shRNA. The expression of MITA was then measured by immunoblot assays. **(C)** EPC cells were transfected with MITA, IRF4b and IRF4b-shRNA, or IRF8 and IRF8-shRNA, and the cells were treated with CHX (100 µg/ml) at 24 h after transfection and lysed every 3 h. The protein level of MITA was examined by immunoblot assays. All the experiments were performed independently at least three times.

To confirm the conclusion above, IRF4b and IRF8 were knocked down for further study. After the knockdown plasmids of IRF4b and IRF8 were successfully constructed, the validity of the two plasmids was verified (**Figure 4A**). The results declared that the protein level of IRF4b decreased gradually with the increasing doses of IRF4b, and the expression of IRF8 presented to be an opposite trend to the increasing doses of IRF8. IRF4b-shRNA, IRF4b, and MITA were then transfected into EPC cells, and EPC cells were also transfected with IRF8-shRNA, IRF8, and MITA to check the protein level of MITA by immunoblot assays (**Figure 4B**). The results illustrated that not only IRF4b -shRNA but also IRF8-shRNA could inhibit the expression of corresponding genes by knocking down, thus affecting the degrees of degradation of MITA. CHX was considered to be a typical inhibitor of protein synthesis. In this study, CHX was used to shorten the half-life time to know the effects of IRF4b or IRF8 on MITA well. Plasmids were transfected into EPC cells as designed in **Figure 4C**. At 24 h after transfection, the cells were treated with CHX and lysed immediately, which was taken as the samples of 0 h, and other samples from the cells were lysed every 3 h. Through interacting with IRF4b, IRF4b-shRNA reduced the protein level of IRF4b, resulting in a decrease in the inhibitory function of IRF4b on the expression of MITA, and it indirectly caused the results that the protein level of MITA was always higher than that in the control group regardless of time changed. Similar results could be obtained when it came to IRF8-shRNA.

### IRF Is the Core Domain of IRF4b and IRF8 in the Regulation of MITA

**Figure 5:**
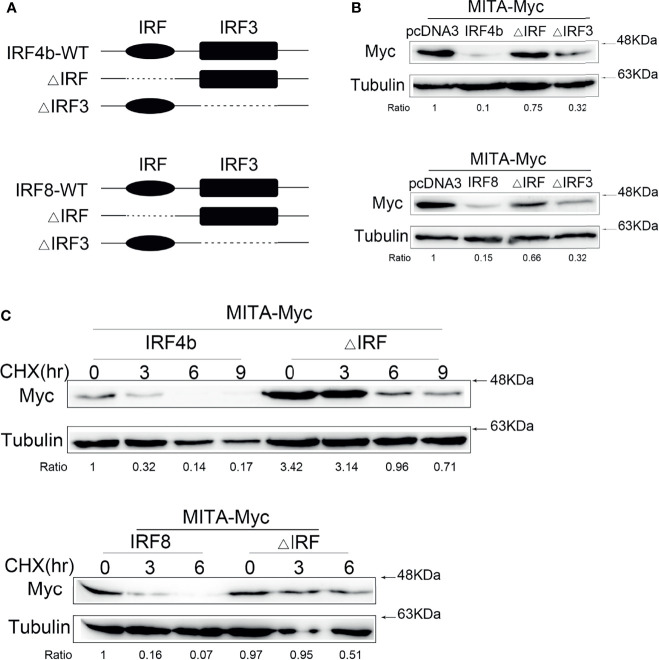
IRF is the core domain of IRF4b and IRF8 in the regulation of MITA. **(A)** Schematic diagrams of the wild type (WT) and mutants of IRF4b or IRF8. **(B)** IRF4b and the mutants of IRF4b were transfected with MITA into EPC cells, and the dose of each plasmid was 0.4 μg (above). MITA was checked by immunoblot assays after 24 h posttransfection, and similar experiments were performed with IRF8, the mutants of IRF8 and MITA (below). **(C)** The IRF-related mutant of IRF4b or IRF8 and IRF4b or IRF8 was transfected with MITA into EPC cells, and CHX was added at 24 h posttransfection. The cells were lysed at different time points and immunoblot assays were conducted. All the experiments were performed independently at least three times.

IRF4b and IRF8 common contain interferon regulatory factor (IRF) and interferon regulatory factor 3 (IRF3) domain, so two mutants of the two genes were constructed, respectively (**Figure 5A**). In order to understand the domain through which IRF4b and IRF8 inhibited the expression of MITA, MITA, IRF4b and its mutants, and IRF8 and its mutants were transfected into EPC cells for immunoblot assays, as shown in **Figure 5B**. In terms of the protein levels of MITA, the presence of IRF4b could promote the degradation of MITA, but when it came to IRF4bΔIRF, the degradation of MITA slowed down. Likewise, it was illustrated again that IRF8 could facilitate the degradation of MITA, and the protein level of MITA in the presence of IRF8ΔIRF was significantly higher than that of MITA in the presence of IRF8. Put another way, it was extremely possible that both IRF4b and IRF8 could accelerate the degradation of MITA through the core domain of IRF. To verify whether IRF was the key domain of these regulatory functions, CHX was added into the cells at 24 h after the plasmids were transfected into EPC cells, and the cells were lysed every 3 h to examine the protein level of MITA (**Figure 5C**). It could be seen in **Figure 5C** that the protein level of MITA continuously decreases as time flew, and the protein level of MITA in the presence of IRF4bΔIRF or IRF8ΔIRF was always higher than that in the presence of IRF4b or IRF8. That was to say, what mattered in the regulatory functions on MITA was the IRF domain of IRF4b or IRF8.

### IRF4b and IRF8 Promote MITA Degradation Through Proteasome Pathway

**Figure 6:**
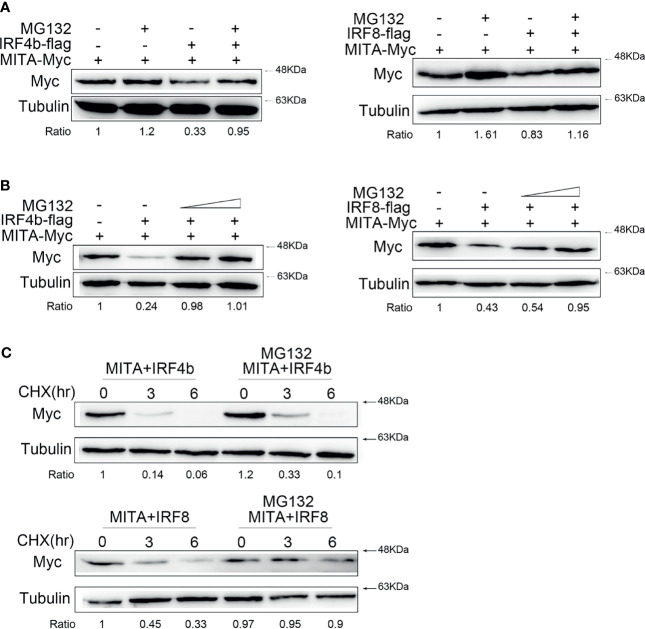
IRF4b and IRF8 promote MITA degradation through proteasome pathway. **(A)** EPC cells were seeded on 12-well plates and transfected with 0.4 μg of MITA and IRF4b or IRF8 for 24 h, and then the cells were treated with DMSO or 30 µM MG132 for 10 h. The expression of MITA was determined by immunoblot assays. **(B)** EPC cells were transfected with 0.4 μg of MITA, IRF4b, or IRF8 again, and increasing doses of MG132 (15 and 30 µM) were added to the cells for 12 h. The expression of MITA was checked by immunoblot assays and normalized to tubulin. **(C)** EPC cells were transfected with 0.4 μg of MITA and IRF4b or IRF8 as designed in **(C)**. At 24 h after transfection, MG132 (30 µM) and CHX (100 µg/ml) were added into cells, and cells were lysed at different time points. The protein level of MITA was examined by immunoblot assays. All the experiments were performed independently at least three times.

It was known to all that the degradation of protein was mainly in three ways. After a large amount of pre-experiments, it was roughly confirmed that the degradation of MITA under the control of IRF4b or IRF8 was through the proteasome pathway. To verify whether the degradation of MITA which was influenced by IRF4b or IRF8 was through the proteasome pathway, MG132, a proteasome inhibitor, was used for more detailed studies. IRF4b or IRF8 was transfected with MITA, and the cells were treated with MG132 in the experimental group while the cells in the control group were treated with the same dose of DMSO to make the balance (**Figure 6A**). When IRF4b or IRF8 existed, the degradation of MITA could be blocked with the participation of MG132, and it was more obvious when the concentration gradient of MG132 was put in use (**Figure 6B**). The results above illustrated that it was through the proteasome pathway that IRF4b and IRF8 promoted the degradation of MITA. To further confirm this, the cells were treated with CHX together with MG132 for immunoblot assays (**Figure 6C**). Not only was the protein level of MITA in the presence of IRF4b gradually decreased by the time but the protein level of MITA in the presence of IRF8 was also going down as time passed by. Moreover, with the addition of MG132, the degradation of MITA was prevented whether in the presence of IRF4b or IRF8.

### IRF4 and IRF8 Lead to the Elevation of MITA Polyubiquitination and Shorten Its Half-Life

**Figure 7:**
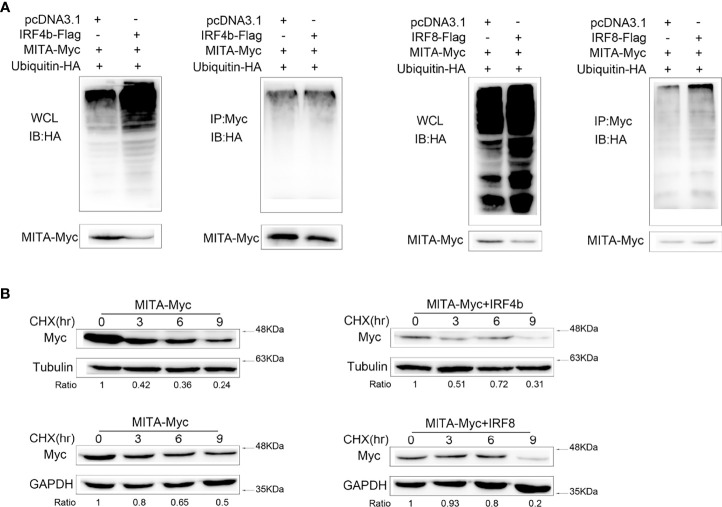
IRF4 and IRF8 lead to the elevation of MITA polyubiquitination and shorten its half-life. **(A)** HEK293 cells were seeded on 10 cm^2^ dishes and cotransfected with MITA, ubiquitin-HA, and IRF4b or IRF8, and the plasmids were all used in 2 μg doses except for ubiquitin-HA which was in 1 μg. At 24 h posttransfection, the cells were treated with MG132 for 10 h, and the cells were lysed to conduct IP assays with Myc antibody and immunoblot assays with antibody against HA. The samples from the immunoprecipitates and whole cell lysates (WCL) were analyzed by immunoblot assays, taking the results from WCL as controls. **(B)** MITA and IRF4b or IRF8 were transfected into EPC cells which were treated with CHX (100 µg/ml) at 24 h after transfection. The dose was always 0.4 μg per plasmid. After cells were lying at different time points, immunoblot assays were performed to examine the expression of MITA. All the experiments were performed independently at least three times.

It could be concluded in **Figure 6** that the protein degradation of MITA induced by IRF4b or IRF8 was possible in the ubiquitin-proteasome pathway. To know whether IRF4b or IRF8 could promote the polyubiquitination of MITA, the plasmids of ubiquitin, MITA, IRF4b, or IRF8 were transfected into the cells, and the cells were then lysed for IP with an antibody against Myc-MITA (**Figure 7A**). The results obtained from immunoblot assays with anti-HA antibody showed that the ubiquitinated MITA in the whole cell lysis with IRF4b or IRF8 was always more than that with empty vector, and similar results were obtained in the cells. The aforesaid results illustrated that IRF4b or IRF8 could accelerate the polyubiquitination of MITA, and the degradation of MITA caused by IRF4b or IRF8 was due to the polyubiquitination. EPC cells were treated with CHX 24 h after transfection and lysed at different time points. Immunoblot analyses were then performed, and the results showed that compared with empty vector transfected cells, the level of MITA was significantly reduced in the presence of IRF4b or IRF8 (**Figure 7B**). These results also manifested that the expression of IRF4b or IRF8 could lead to the increase of MITA polyubiquitination.

## Discussion

NF-κB was a fast-response transcription factor that existed in almost all cells to regulate the transcription of a great deal of genes. What is more, NF-κB could participate in biological processes such as inflammatory response, immune response, apoptosis, and so on through the cytokines that are regulated by NF-kB. As a result, it could participate in many signaling pathways and play an important role in the normal physiological process and the occurrence of diseases (24, 28, 29). Many diseases caused by inflammation were related to overactivation of NF-κB, and studies had found that noncoding genes had an impact on NF-κB signaling pathway. MiR-3570 could directly target the 3′UTR region of MyD88 and affect NF-κB signaling pathway through posttranscriptional regulation, and miR-214 was also found to have a similar function as miR-3570 (30, 31) with the exception that some miRNAs made direct functions on NF-κB pathway by inhibiting the expression of the subunit of NF-κB called p65 (32). In addition, coding genes still work. For example, NLRX1 has a negative reaction on TRAF6-induced NF-κB signaling pathway (5). The overexpression of USP2a leads to a form of deubiquitinated TRAF6, and it could inhibit the activation of NF-κB and the transcription of inflammatory cytokines (33). The activation of NF-κB induced by TLRs was indirectly inhibited by WWP2 and TRIM38 *via* targeting different genes for ubiquitination and degradation (34, 35). In this study, IRF4b and IRF8 were found to have inhibitory function on MITA-mediated NF-κB signaling pathway, which provides evidence on the impacts of NF-κB signaling pathway caused by coding genes.

In the innate immune responses, evidence shows that the members of interferon regulatory factors (IRFs) could participate in the regulations of the signaling pathways related to immunity and the expression of IFN (36). As known to all, MyD88 played a crucial role in TLR-mediated NF-κB signaling pathway. MyD88 could interact with IRF3 and IRF7 and regulate the IRF-induced type I IFN response in Atlantic salmon (37). Also, IRF4 (38) and IRF5 (39) affected the downstream of TLR signaling pathway, and IRF4 could compete with IRF5 for MyD88 interaction. In addition, IRF5 was involved in the regulation of RLR signaling pathway; it was found that the expression level of type I IFNs significantly decreased due to the absence of IRF5. Based on these studies, there was no difficulty to find that the interaction between IRFs and immune molecules was considered an important regulatory mechanism in immune responses. In this study, we found that IRF4b and IRF8 could promote the degradation of MITA and have an inhibitory function on MITA-mediated NF-κB signaling pathway, and the stability of MITA-mediated NF-κB signaling pathway was maintained by IRF4b and IRF8 through the degradation of MITA in the ubiquitin-proteasome pathway.

Except for making functions on the immune-related signaling pathways, IRFs also played a significant role in regulating the expression of IFN genes in immunity (40, 41). IRFs consisted of 11 members in fish and were divided into positive and negative regulators. IRF3 could trigger the expression of IFN while IRF2 and IRF10 could inhibit that (42, 43). In this study, IRF4b and IRF8 were considered negative regulators to have an impact on the degradation of MITA and shared the same mechanism in the regulation of MITA. Both IRF4b and IRF8 could increase the ubiquitination of MITA and promote the degradation of MITA. However, the underlying molecular mechanisms of whether IRF4b and IRF8 have E3 ubiquitin ligase activity or whether they could directly catalyze ubiquitination have not been determined. IRF4b and IRF8 belonging to the same family had the same domains: one was the IRF domain and the other was the IRF3 domain. The IRF domain of IRF4b or IRF8 was found to be crucial to the negative regulation of MITA, which corresponds to the IRF being able to interact with a DNA region which regulated the transcription. Therefore, IRF4b and IRF8 had inhibitory function on MITA-mediated NF-κB signaling pathway.

Generally speaking, the study has identified IRF4b and IRF8 as negative regulators to promote the degradation of MITA through the ubiquitin-proteasome pathway and influence the MITA-mediated signaling pathway. These datums enrich the contents of the RLR signaling pathway and provide new insights into the regulatory mechanism in teleost fish.

## Data Availability Statement

The original contributions presented in the study are included in the article/**Supplementary Material**. Further inquiries can be directed to the corresponding author.

## Ethics Statement

All animal experiments were conducted in accordance with the guidelines for the Care and Use of Experimental Animals issued by the National Institutes of Health, and the experiments were approved by the Research Ethics Committee of Shanghai Ocean University (No. SHOU-DW-2018-047).

## Author Contributions

Conceived and designed the experiments: YS and TX. Performed the experiments: XY, XZ, and MZ. Analyzed the data: TX and XY. Contributed reagents/materials/analysis tools: XY, XZ, and MZ. Wrote the paper: TX and XY. All authors listed have made a substantial, direct, and intellectual contribution to the work and approved it for publication.

## Funding

This study was supported by the National Natural Science Foundation of China (31822057).

## Conflict of Interest

The authors declare that the research was conducted in the absence of any commercial or financial relationships that could be construed as a potential conflict of interest.

## Publisher’s Note

All claims expressed in this article are solely those of the authors and do not necessarily represent those of their affiliated organizations, or those of the publisher, the editors and the reviewers. Any product that may be evaluated in this article, or claim that may be made by its manufacturer, is not guaranteed or endorsed by the publisher.
