# Supplementary material for: IRF4b and IRF8 Negatively Regulate RLR-Mediated NF-κB Signaling by Targeting MITA for Degradation in Teleost Fish
Source: Front Immunol. 2022 Mar 3;13:858179. doi: 10.3389/fimmu.2022.858179 (PMC8927078; doi:10.3389/fimmu.2022.858179)
Supplement: Supplementary file 1 [file DataSheet_1.pdf]

Supplemental Table I The information of the sequences of PCR primers in this study.

**Primers**	**Sequences (5'-3')**
**Vector construction**	
MITA-HindIII-F	CCCAAGCTTCTGTGCCTCCAGGATCAG
MITA-EcoRI-R	CCGGAATTCAGAGCAAGAATGTGGTGT
MITA-GFP-HindIII-F	CCCAAGCTTATGCTGTGCCTCCAGGATCA
MITA-GFP-BamHI-R	CGCGGATCCGCCATAAATCCCTGATAATTGTCGG
IRF4b-HindIII-F	GACGATGACGACAAGAAGCTTAACGCAGACTTGGATTACGGAG
IRF4b-EcoRI-R	TGATGGATATCTGCAGAATTCTCACTCCTGCAGGCTGCTG
IRF4b-shRNA-F	GATCCGCTGGAGAGATTACTGTCATTCAAGAGATGACAGTAATCTCTCCAGCTTTTTTG
IRF4b-shRNA-R	AATTCAAAAAAGCTGGAGAGATTACTGTCATCTCTTGAATGACAGTAATCTCTCCAGCG
IRF4b-dIRF-BamHI-F	CGCGGATCCGGAGCCAAGAAAAGACCCC
IRF4b-dIRF-BamHI-R	CGCGGATCCGGAGCCTCCGTAATCCAAG
IRF4b-dIRF3-BamHI-F	CGCGGATCCCAGATGGGCGGCCACTACTA
IRF4b-dIRF3-BamHI-R	CGCGGATCCTGAGAGCGCCTCCGCTGAT
IRF8-BamHI-F	CGCGGATCCATGTCAAACACGGGAGGTC
IRF8-XbaI-R	TGCTCTAGAAGTTCAGGCAGTGATTGGC
IRF8-dIRF-XhoI-F	CCGCTCGAGGAGGAGCAGAAGAATGGC
IRF8-dIRF-XhoI-R	CCGCTCGAGCCGACCTCCCGTGTTTGACAT
IRF8-dIRF3-XhoI-F	CCGCTCGAGGATGCTGTGAATATGCGC
IRF8-dIRF3-XhoI-R	CCGCTCGAGCATCATTTGGGAGAAAGC
IRF8-shRNA-F	GATCCGCCGCACTTTGTTTCGAATTTCAAGAGAATTCGAAACAAAGTGCGGCTTTTTTG
IRF8-shRNA-R	AATTCAAAAAAGCCGCACTTTGTTTCGAATTCTCTTGAAATTCGAAACAAAGTGCGGCG
**Real-time PCR**	
MITA-RT-F	CAACGCCAACATTTCTCAC
MITA-RT-R	ACGCTGTGCTTGTAGACCC
IRF4b-RT-F	TGGGAGAACGATGAGAAGA
IRF4b-RT-R	GGGATGATGCGGTACACT
IRF-8-RT-F	ACACGCAGGGAAACAGGATT
IRF-8-RT-R	GCACAGCGGAGTCTGGTCTT
β-actin-RT-F	GAGCCGCACGCTTCTTT
β-actin-RT-R	CTGCTGTAGCCGAGGAC
