# Supplementary figures and images for: IRF4b and IRF8 Negatively Regulate RLR-Mediated NF-κB Signaling by Targeting MITA for Degradation in Teleost Fish
Source: Front Immunol. 2022 Mar 3;13:858179. doi: 10.3389/fimmu.2022.858179 (PMC8927078; doi:10.3389/fimmu.2022.858179)

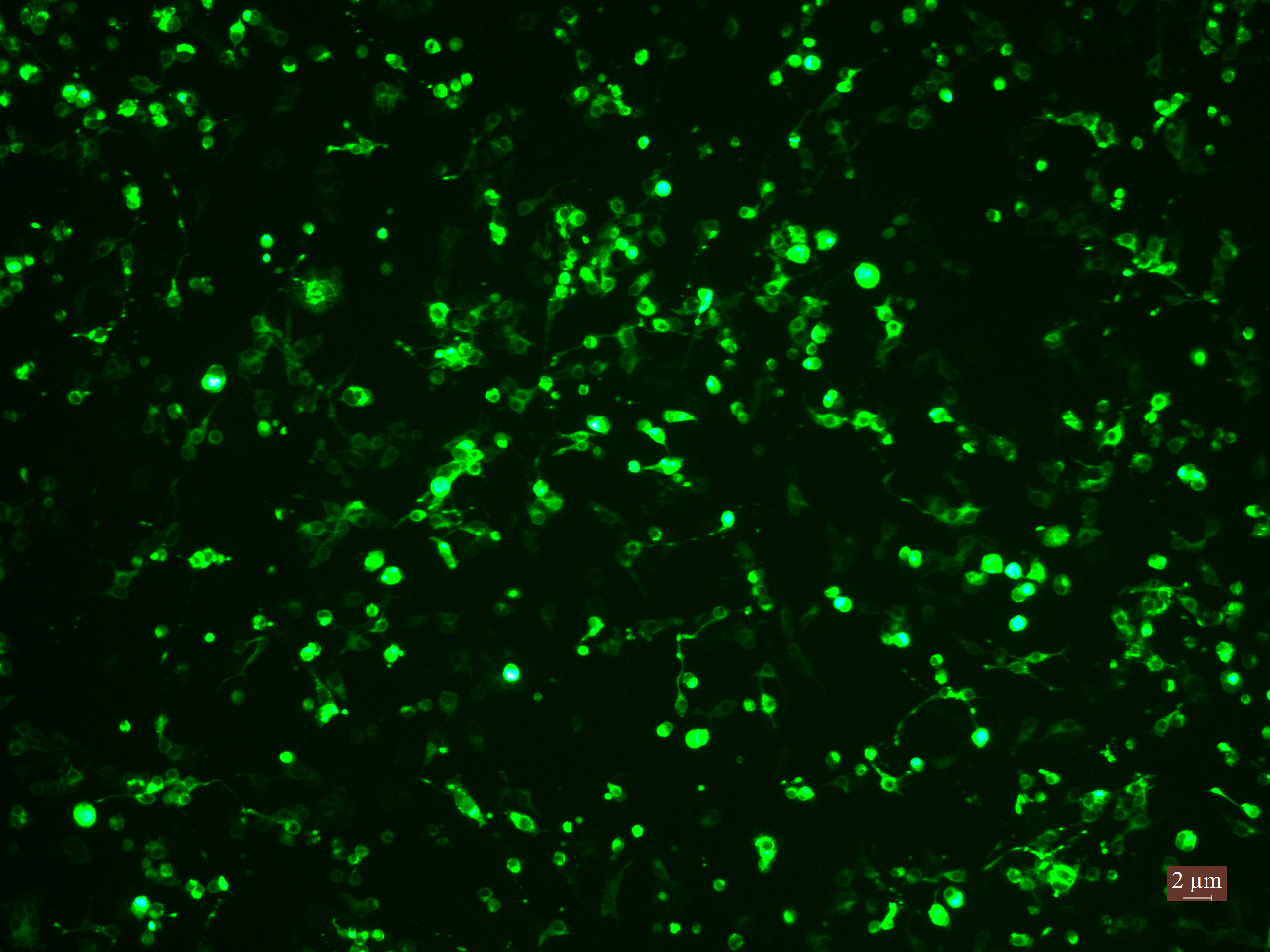

Supplement: Supplementary file 2 [file DataSheet_2.zip › Source data/GFP fluorescence image/Fig. 3E-bottom/Fig. 3E—MITA-GFP+IRF8-Flag.tif]

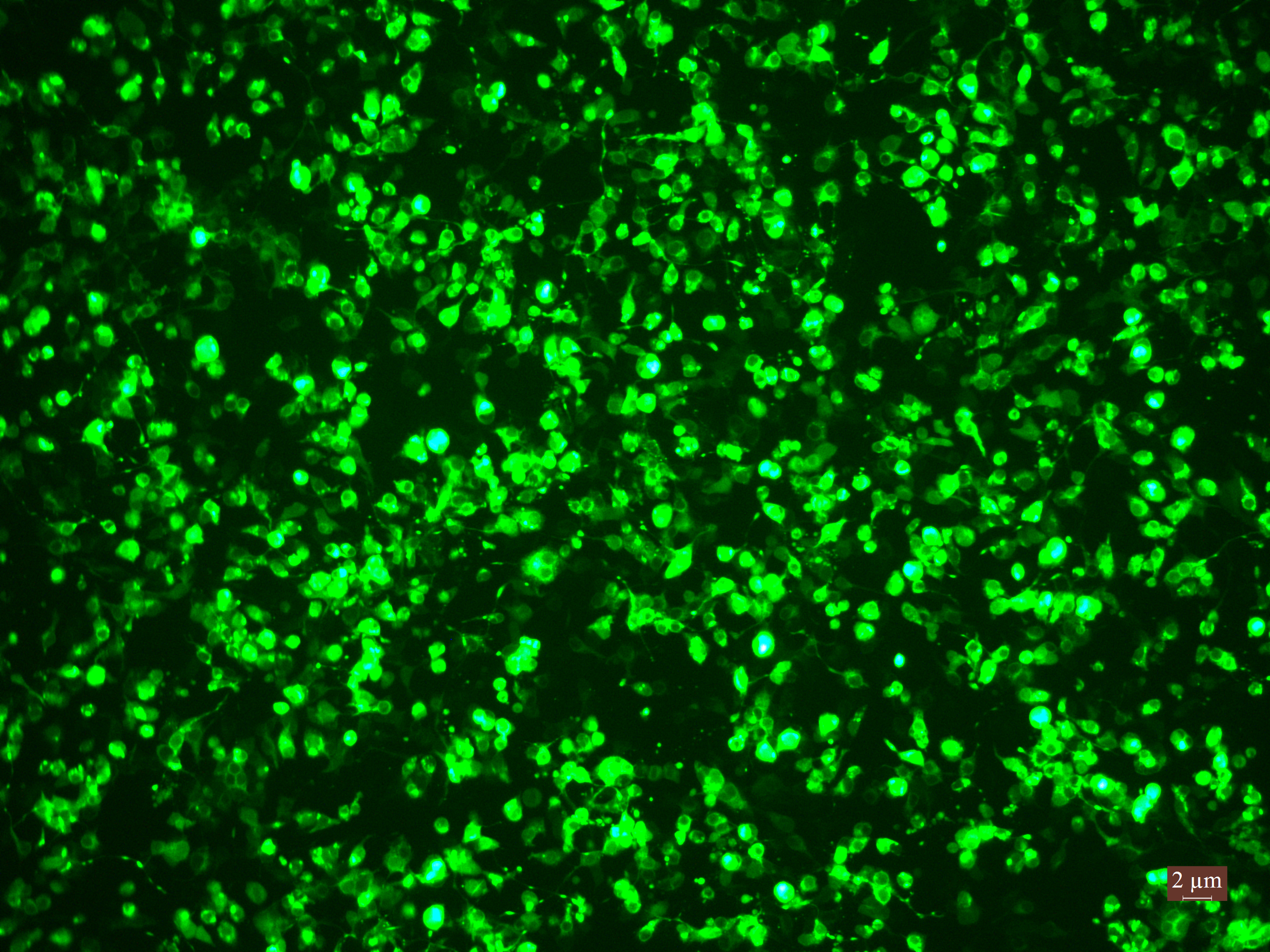

Supplement: Supplementary file 2 [file DataSheet_2.zip › Source data/GFP fluorescence image/Fig. 3E-bottom/Fig. 3E—MITA-GFP+pcDNA3.1.tif]

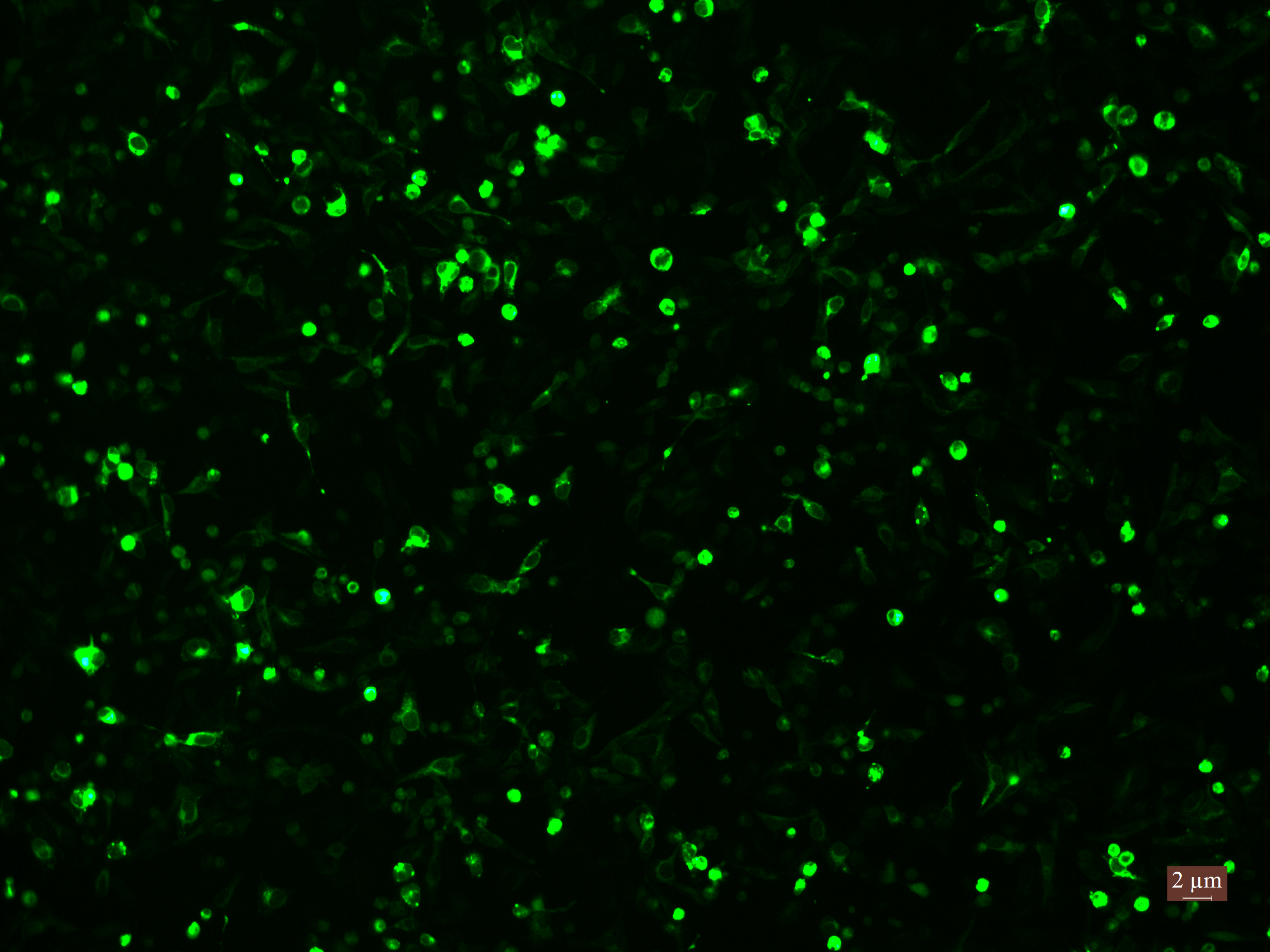

Supplement: Supplementary file 2 [file DataSheet_2.zip › Source data/GFP fluorescence image/Fig. 3E-top/Fig. 3E—MITA-GFP+IRF4b-Flag.tif]

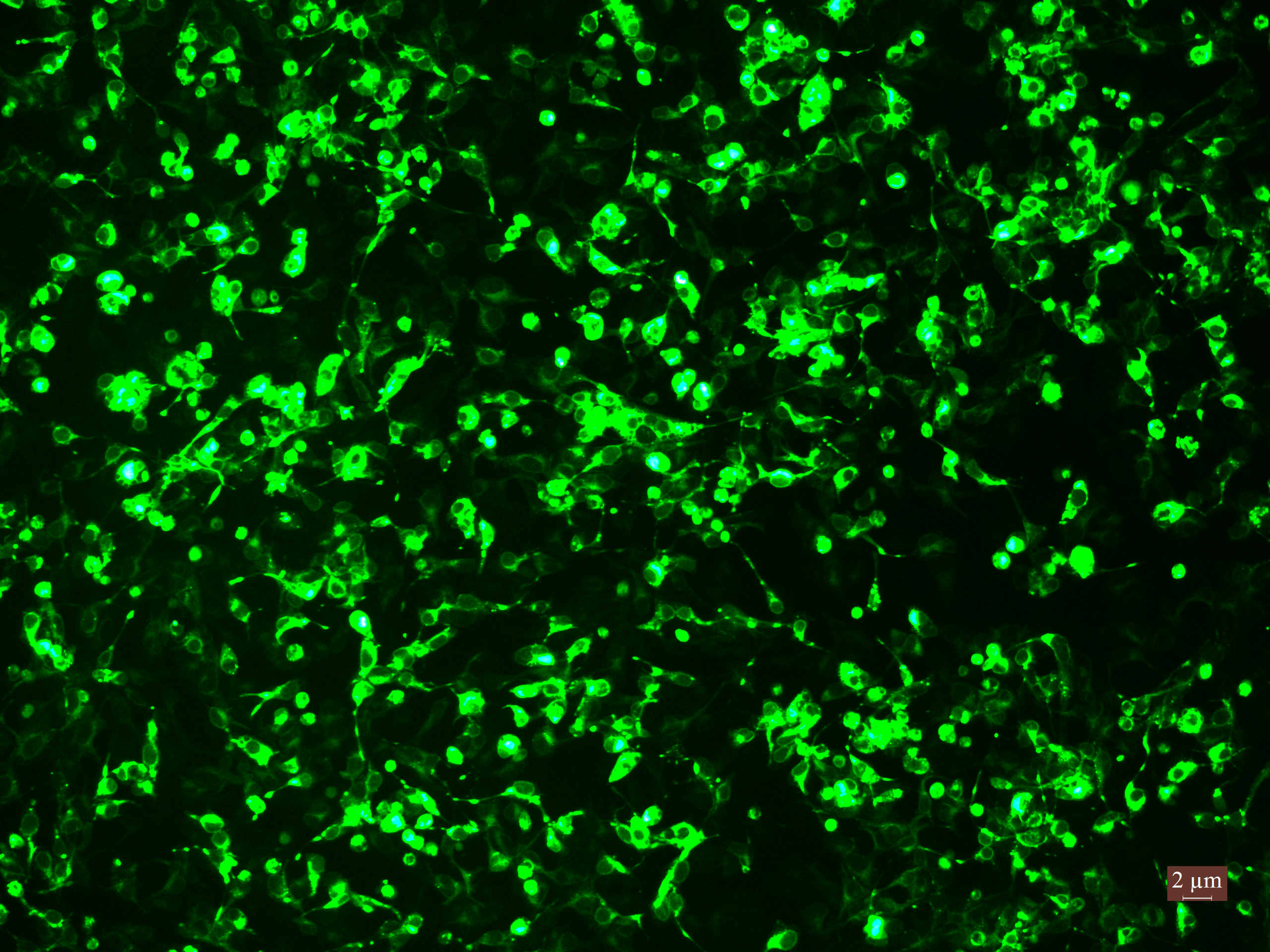

Supplement: Supplementary file 2 [file DataSheet_2.zip › Source data/GFP fluorescence image/Fig. 3E-top/Fig. 3E—MITA-GFP+pcDNA3.1.tif]

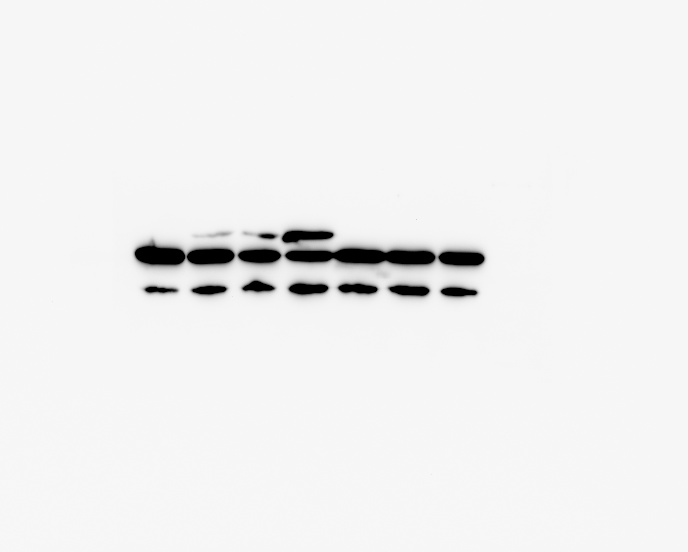

Supplement: Supplementary file 2 [file DataSheet_2.zip › Source data/Western Blot gel scan image/Fig. 3A/Fig. 3A-left—anti-GAPDH.tif]

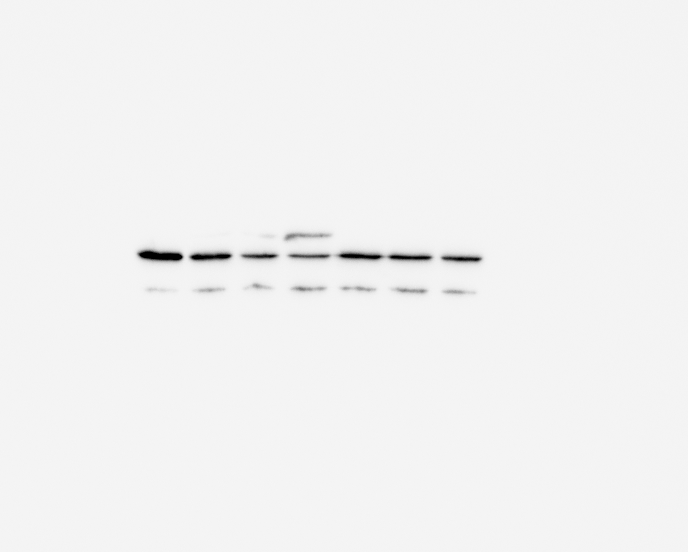

Supplement: Supplementary file 2 [file DataSheet_2.zip › Source data/Western Blot gel scan image/Fig. 3A/Fig. 3A-left—anti-Myc.tif]

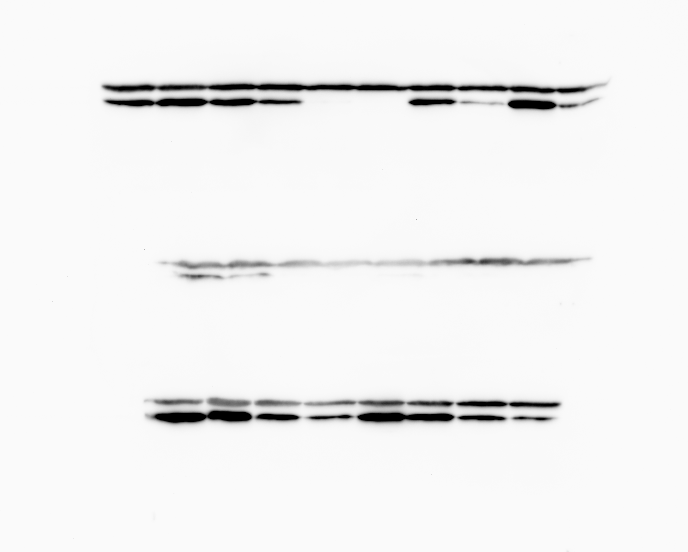

Supplement: Supplementary file 2 [file DataSheet_2.zip › Source data/Western Blot gel scan image/Fig. 3A/Fig. 3A-right—anti-Myc+Tubulin.tif]

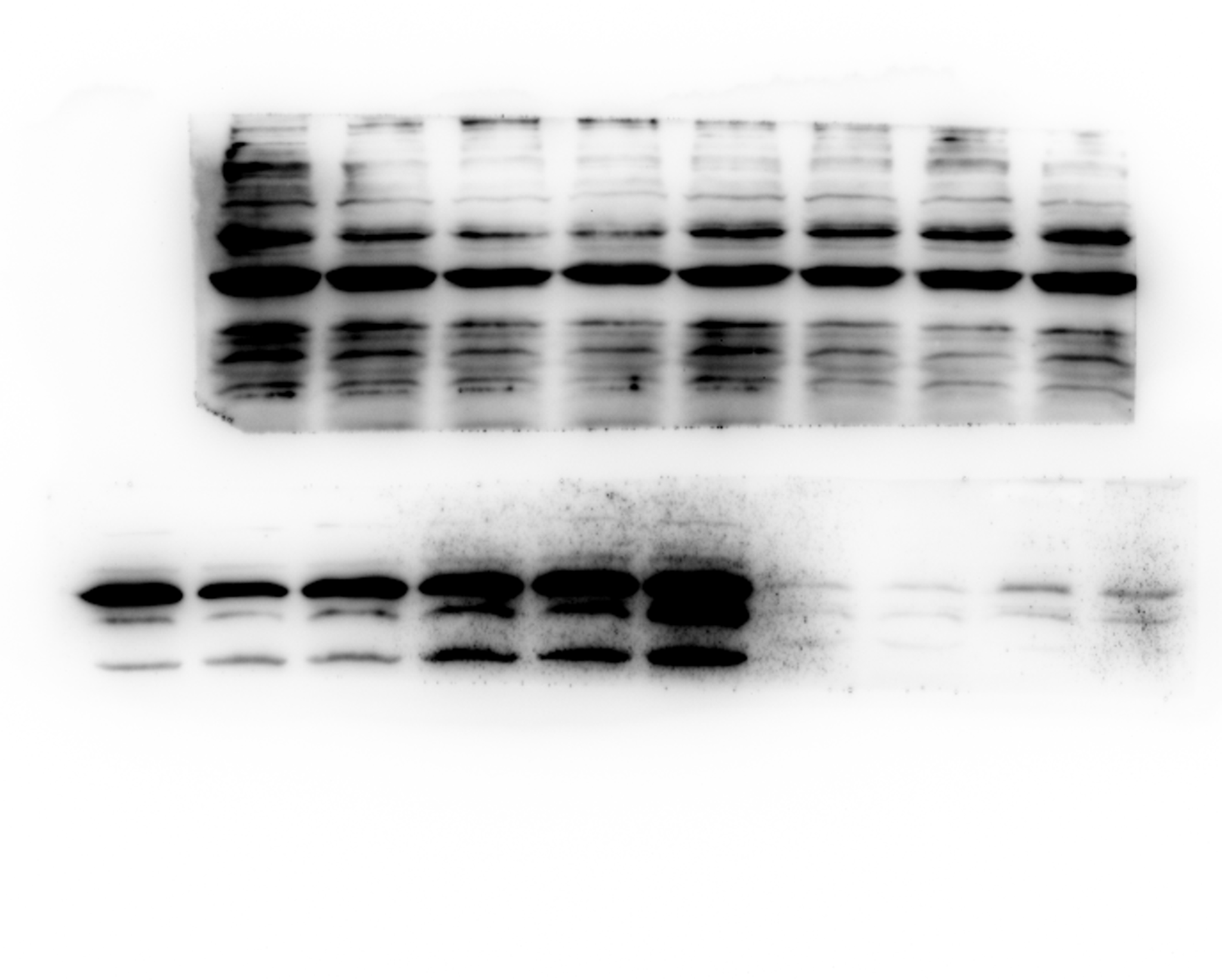

Supplement: Supplementary file 2 [file DataSheet_2.zip › Source data/Western Blot gel scan image/Fig. 3B/Fig. 3B—anti-MITA.Tif]

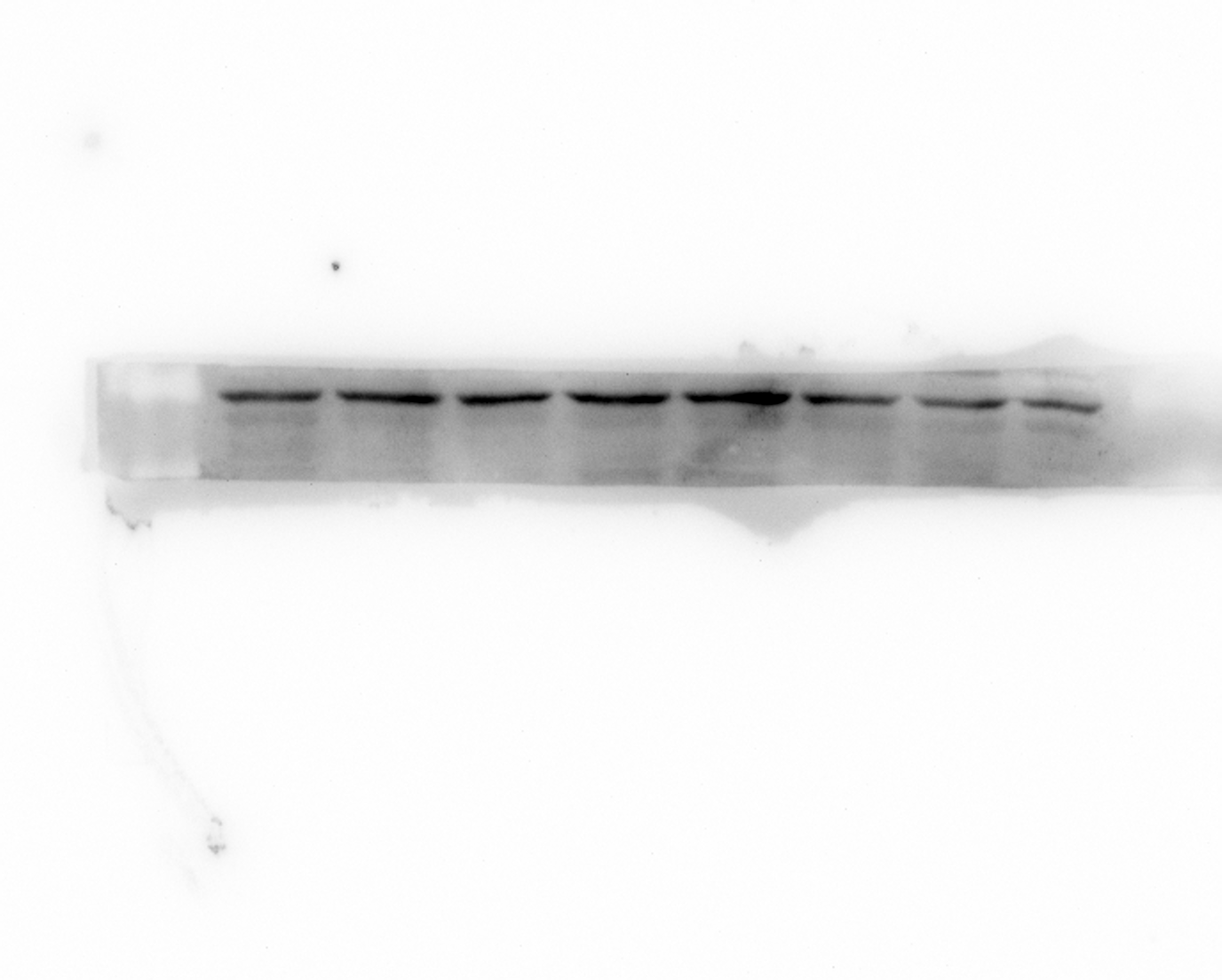

Supplement: Supplementary file 2 [file DataSheet_2.zip › Source data/Western Blot gel scan image/Fig. 3B/Fig. 3B—anti-Tubulin.Tif]

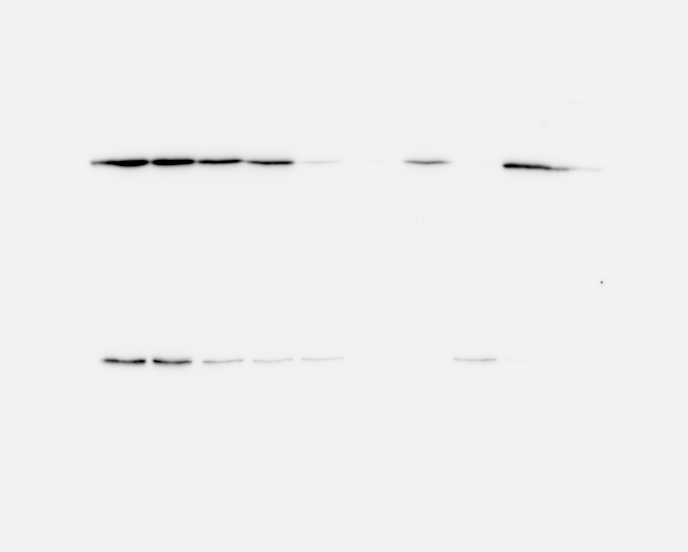

Supplement: Supplementary file 2 [file DataSheet_2.zip › Source data/Western Blot gel scan image/Fig. 3C/Fig. 3C-left—anti-Myc.tif]

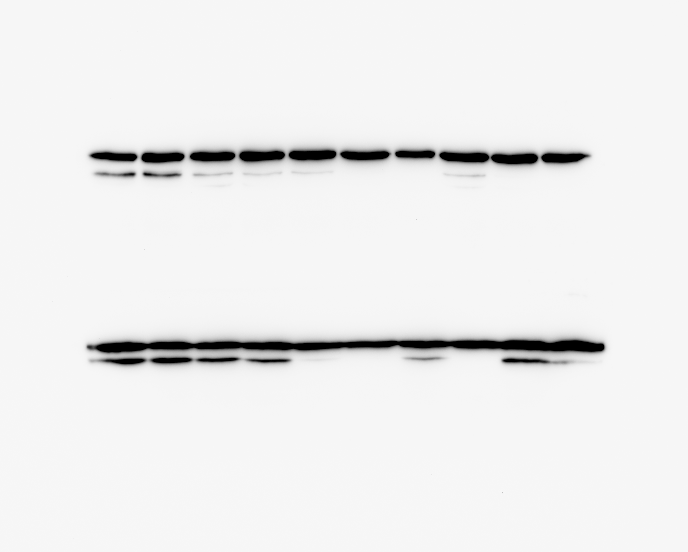

Supplement: Supplementary file 2 [file DataSheet_2.zip › Source data/Western Blot gel scan image/Fig. 3C/Fig. 3C-left—anti-Tubulin.tif]

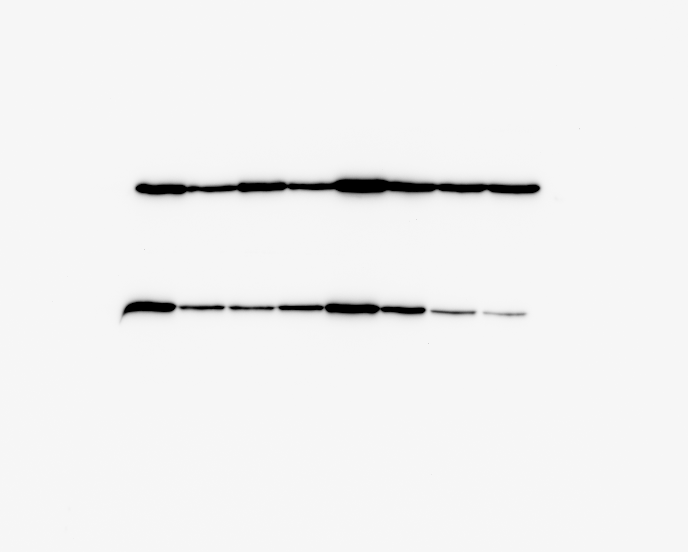

Supplement: Supplementary file 2 [file DataSheet_2.zip › Source data/Western Blot gel scan image/Fig. 3C/Fig. 3C-right—anti-Myc.tif]

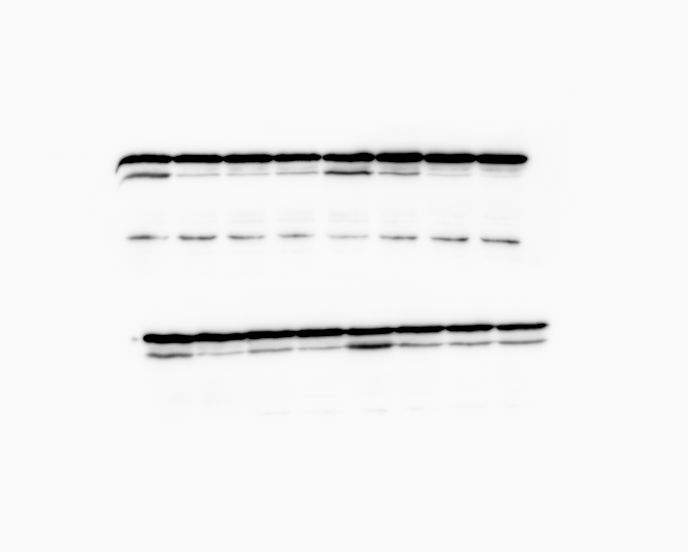

Supplement: Supplementary file 2 [file DataSheet_2.zip › Source data/Western Blot gel scan image/Fig. 3C/Fig. 3C-right—anti-Tubulin.tif]

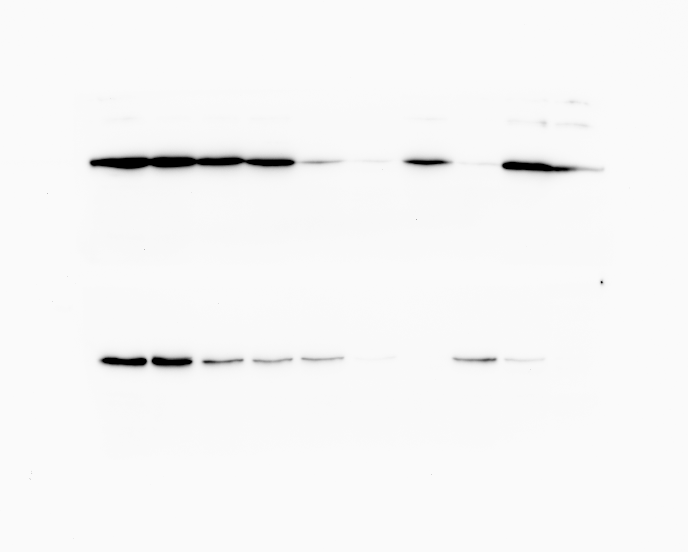

Supplement: Supplementary file 2 [file DataSheet_2.zip › Source data/Western Blot gel scan image/Fig. 3D/Fig. 3D-left—anti-Myc.tif]

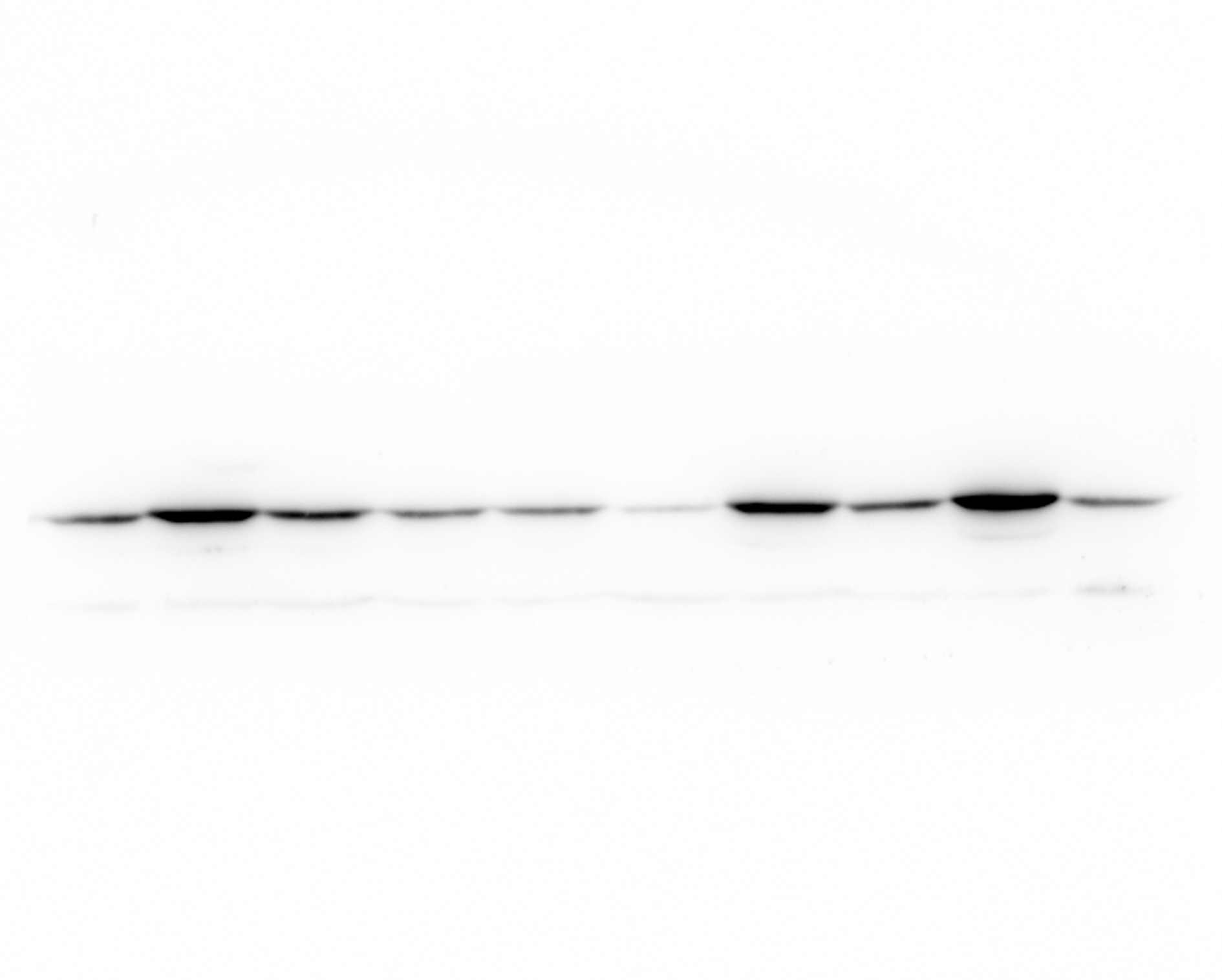

Supplement: Supplementary file 2 [file DataSheet_2.zip › Source data/Western Blot gel scan image/Fig. 3D/Fig. 3D-right—anti-Myc.Tif]

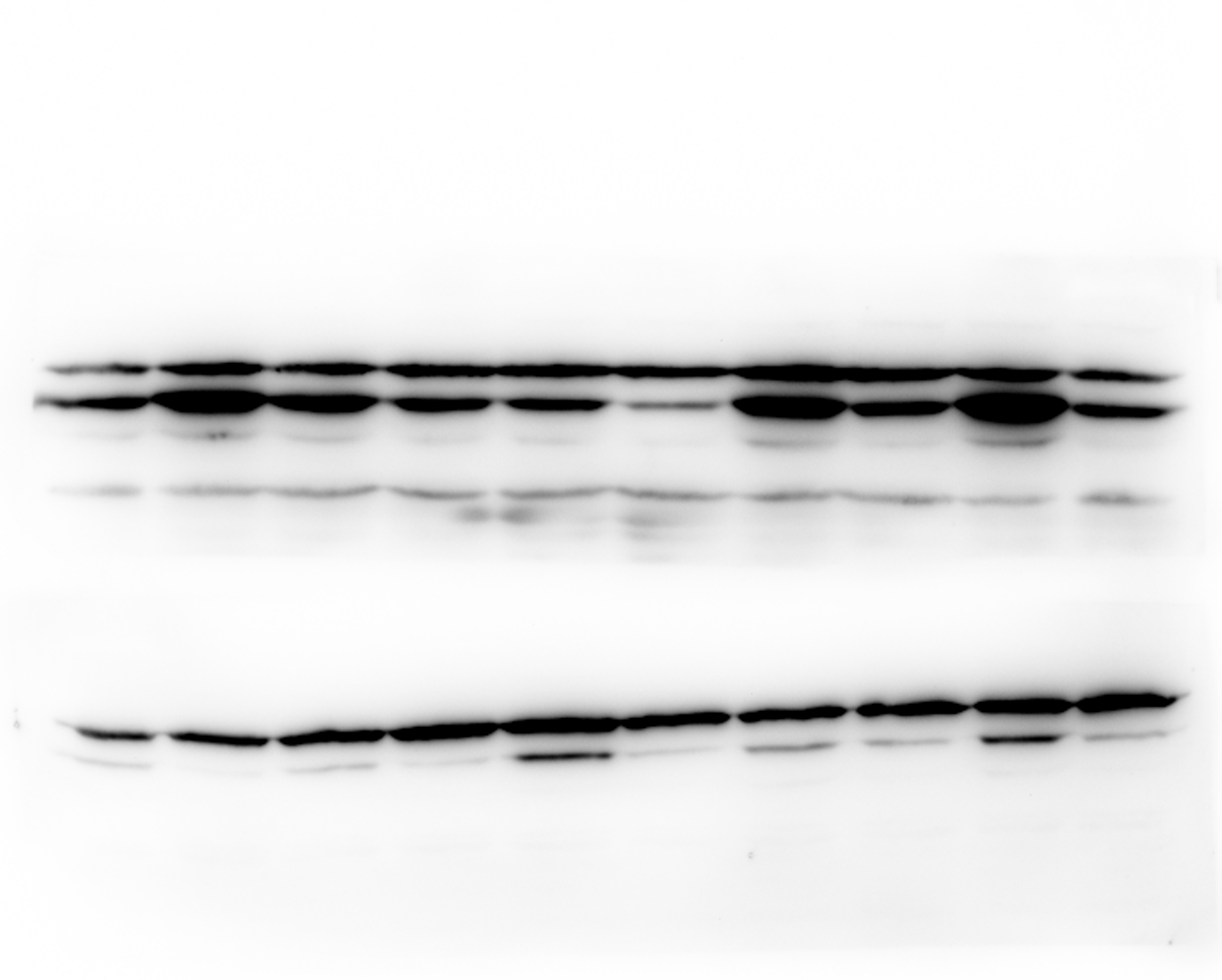

Supplement: Supplementary file 2 [file DataSheet_2.zip › Source data/Western Blot gel scan image/Fig. 3D/Fig. 3D-right—anti-Tubulin.Tif]

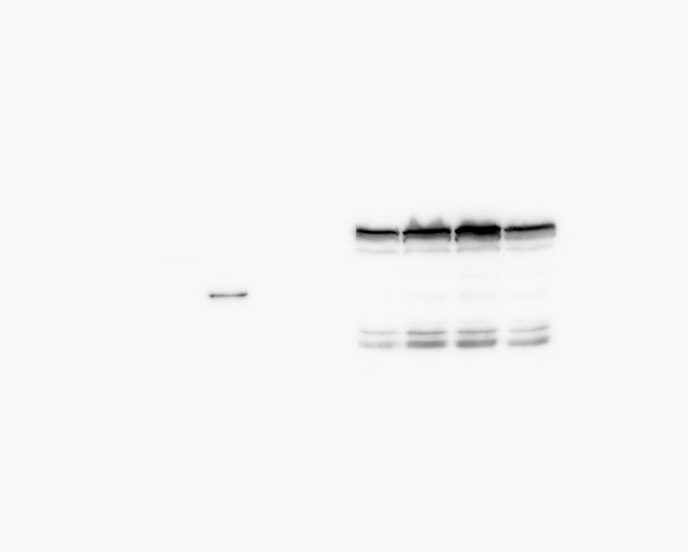

Supplement: Supplementary file 2 [file DataSheet_2.zip › Source data/Western Blot gel scan image/Fig. 3E/Fig. 3E-bottom—anti-GFP.tif]

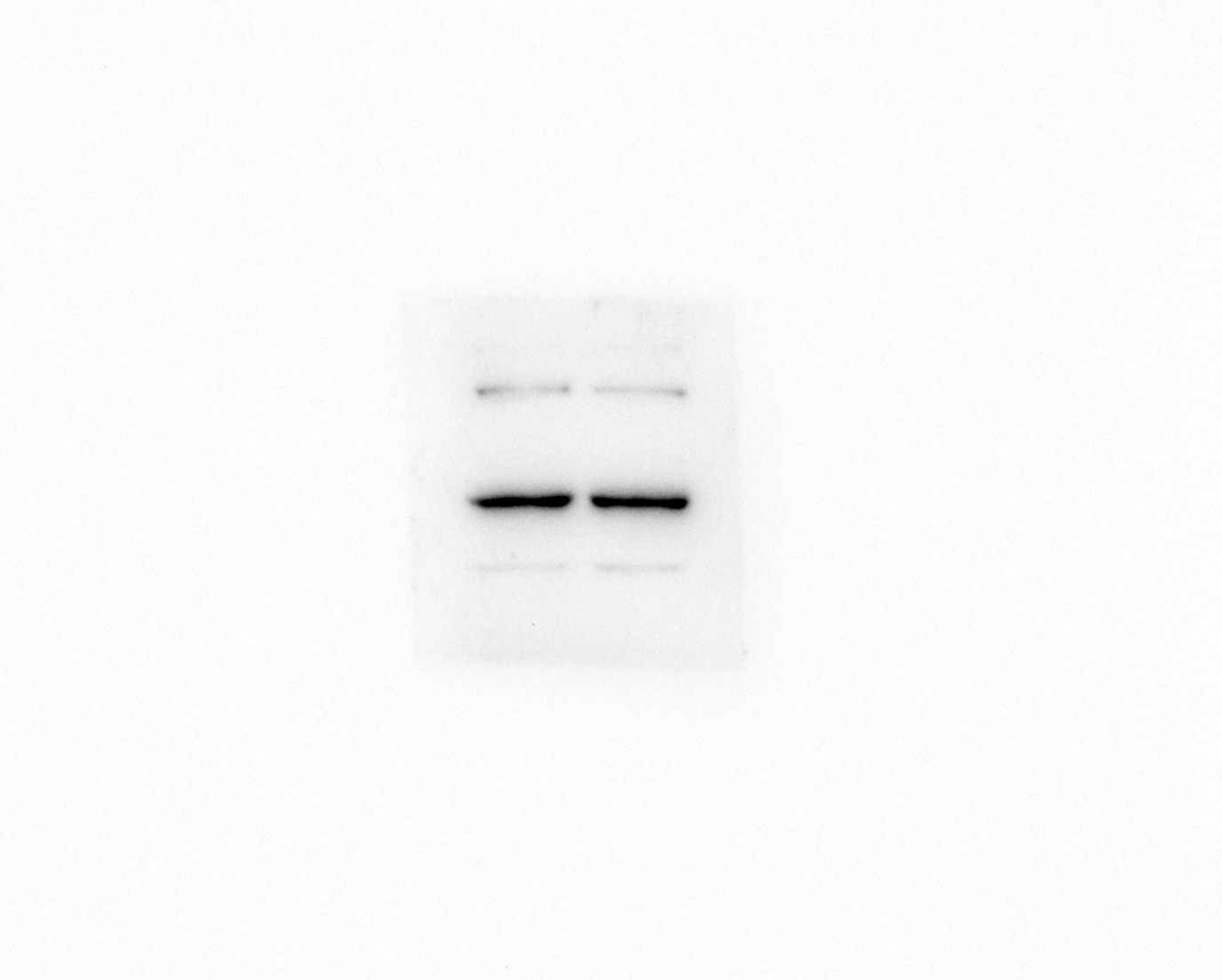

Supplement: Supplementary file 2 [file DataSheet_2.zip › Source data/Western Blot gel scan image/Fig. 3E/Fig. 3E-bottom—anti-Tubulin.tif]

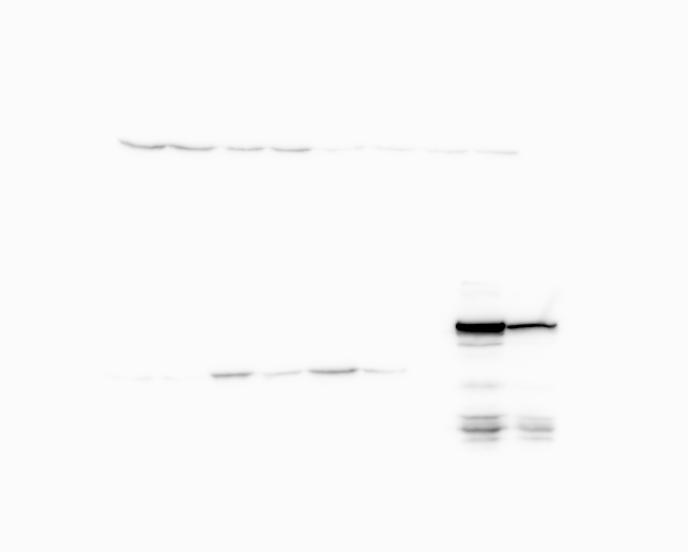

Supplement: Supplementary file 2 [file DataSheet_2.zip › Source data/Western Blot gel scan image/Fig. 3E/Fig. 3E-top—anti-GFP.tif]

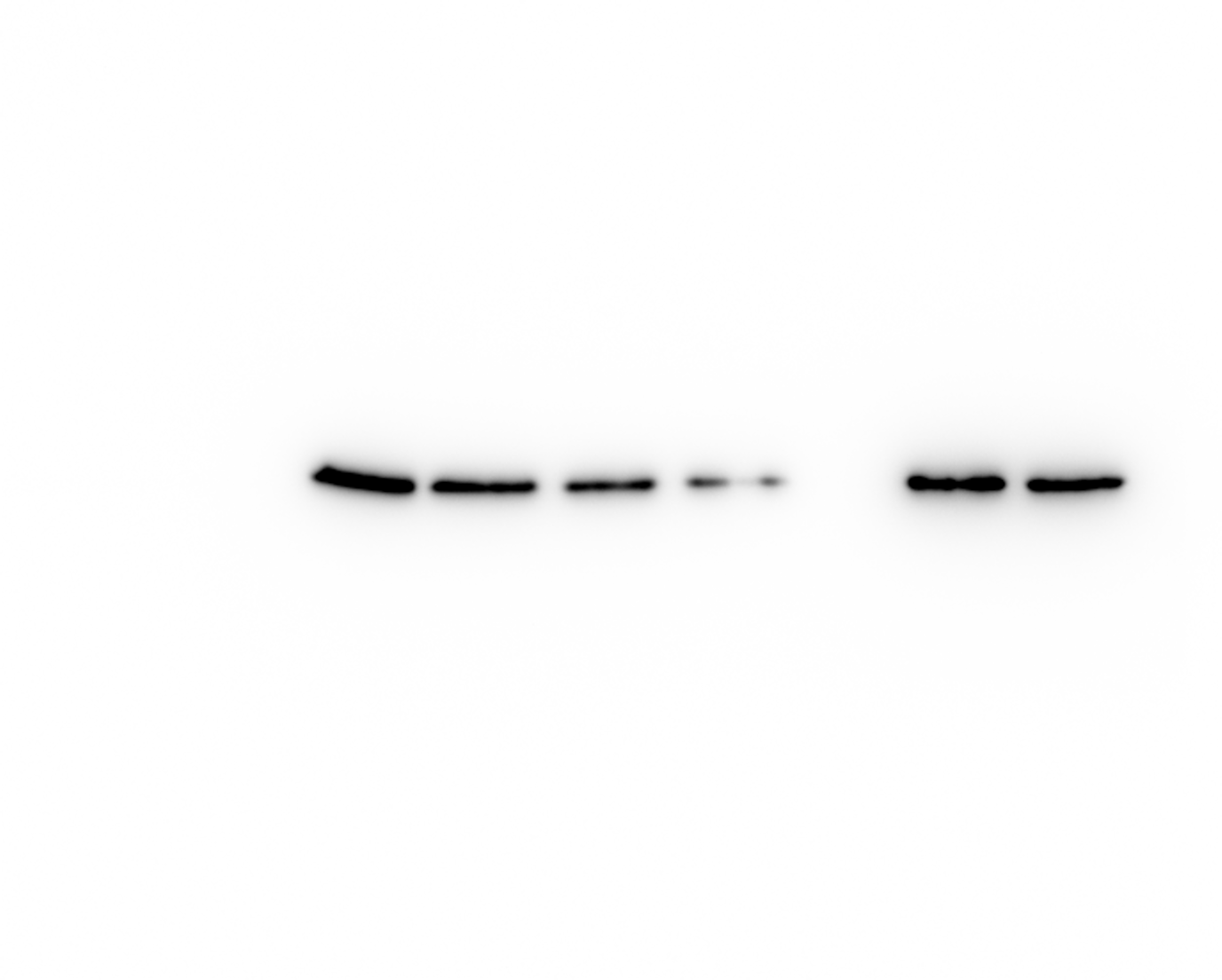

Supplement: Supplementary file 2 [file DataSheet_2.zip › Source data/Western Blot gel scan image/Fig. 3E/Fig. 3E-top——anti-GAPDH.tif]

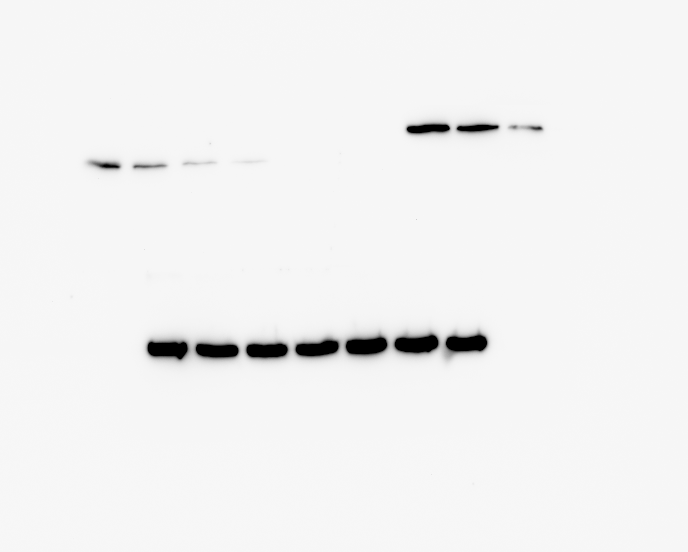

Supplement: Supplementary file 2 [file DataSheet_2.zip › Source data/Western Blot gel scan image/Fig. 4A/Fig. 4A-left—anti-Flag.tif]

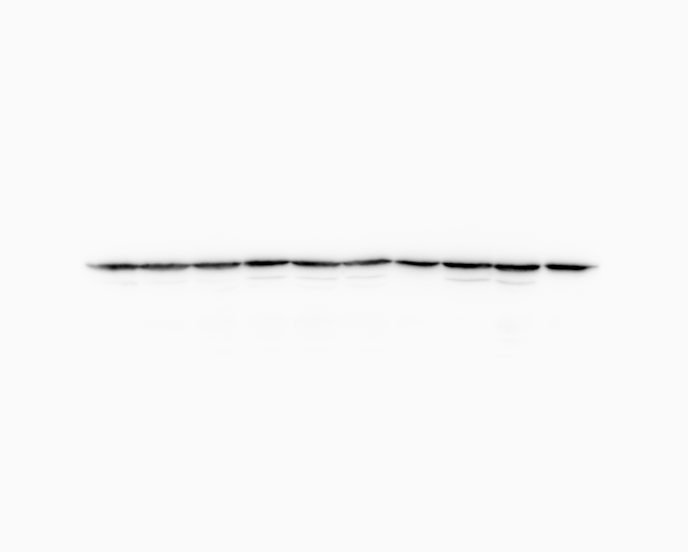

Supplement: Supplementary file 2 [file DataSheet_2.zip › Source data/Western Blot gel scan image/Fig. 4A/Fig. 4A-left—anti-Tubulin.tif]

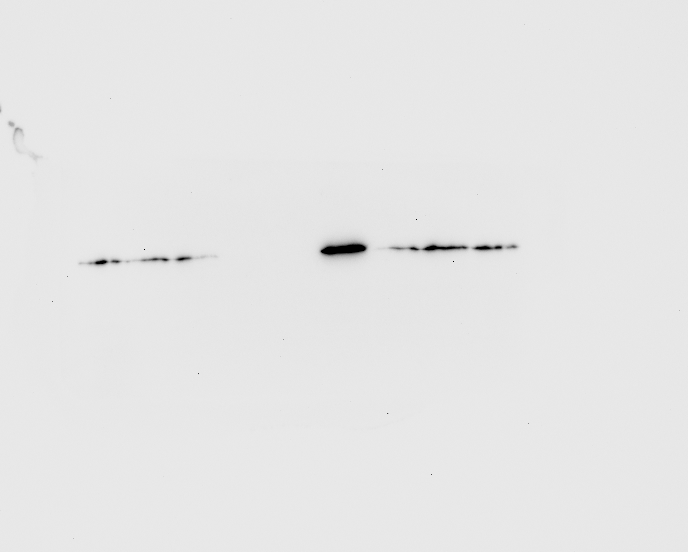

Supplement: Supplementary file 2 [file DataSheet_2.zip › Source data/Western Blot gel scan image/Fig. 4A/Fig. 4A-right—anti-Flag.tif]

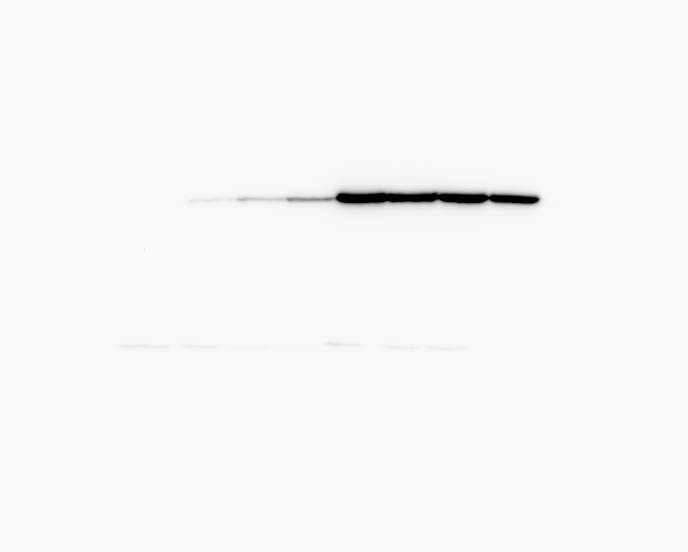

Supplement: Supplementary file 2 [file DataSheet_2.zip › Source data/Western Blot gel scan image/Fig. 4A/Fig. 4A-right—anti-Tubulin.tif]

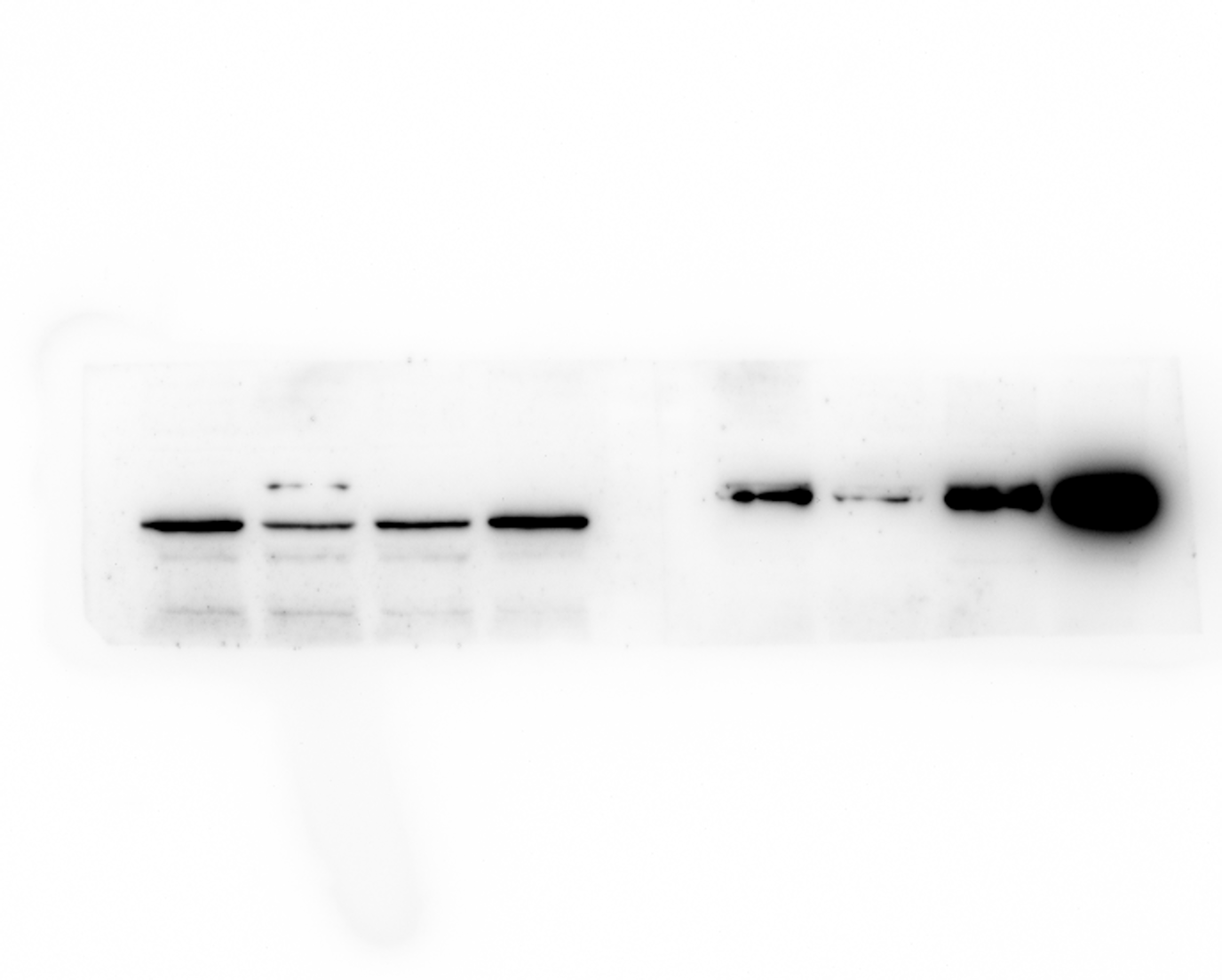

Supplement: Supplementary file 2 [file DataSheet_2.zip › Source data/Western Blot gel scan image/Fig. 4B/Fig. 4B-left—anti-Myc.Tif]

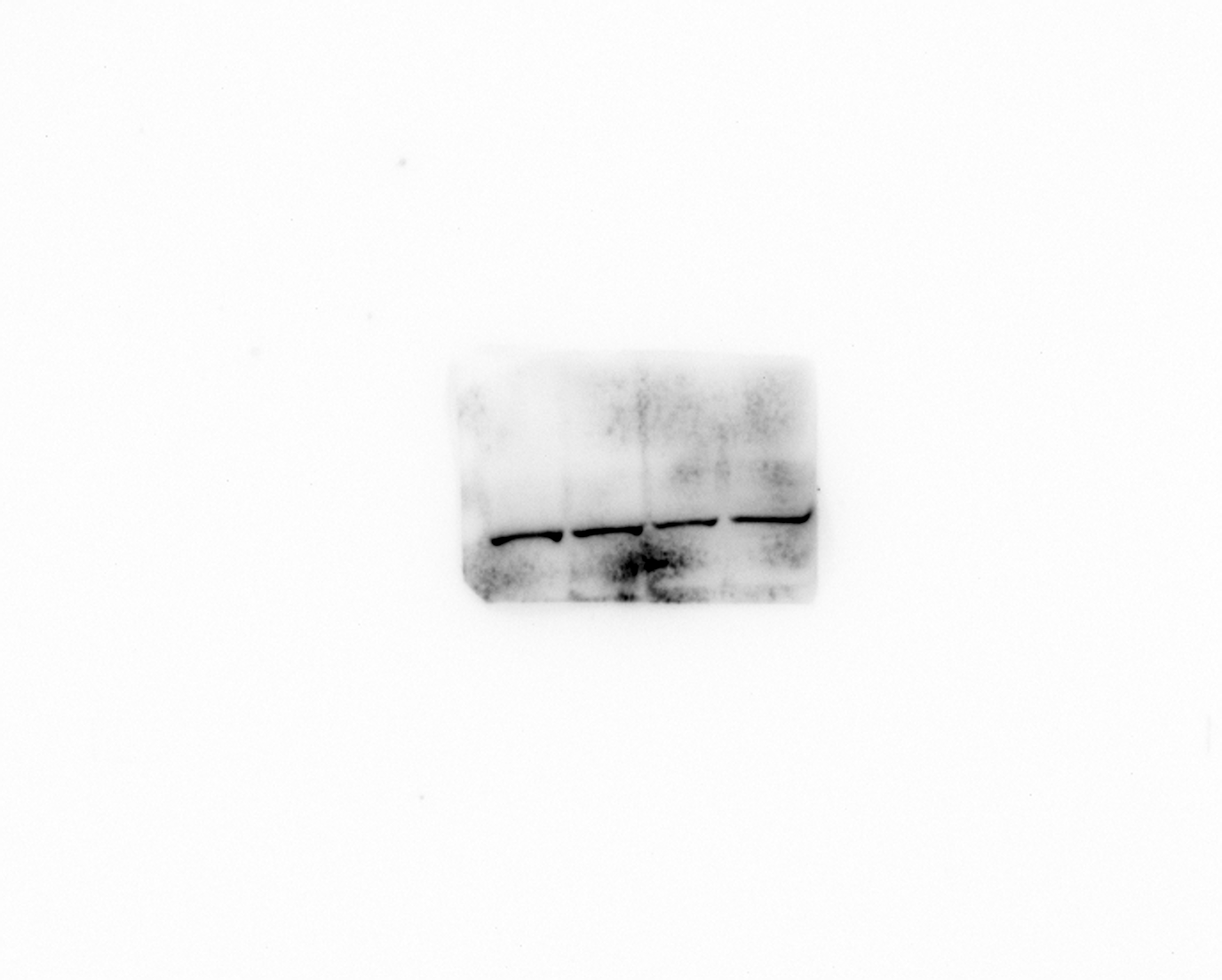

Supplement: Supplementary file 2 [file DataSheet_2.zip › Source data/Western Blot gel scan image/Fig. 4B/Fig. 4B-left—anti-Tubulin.Tif]

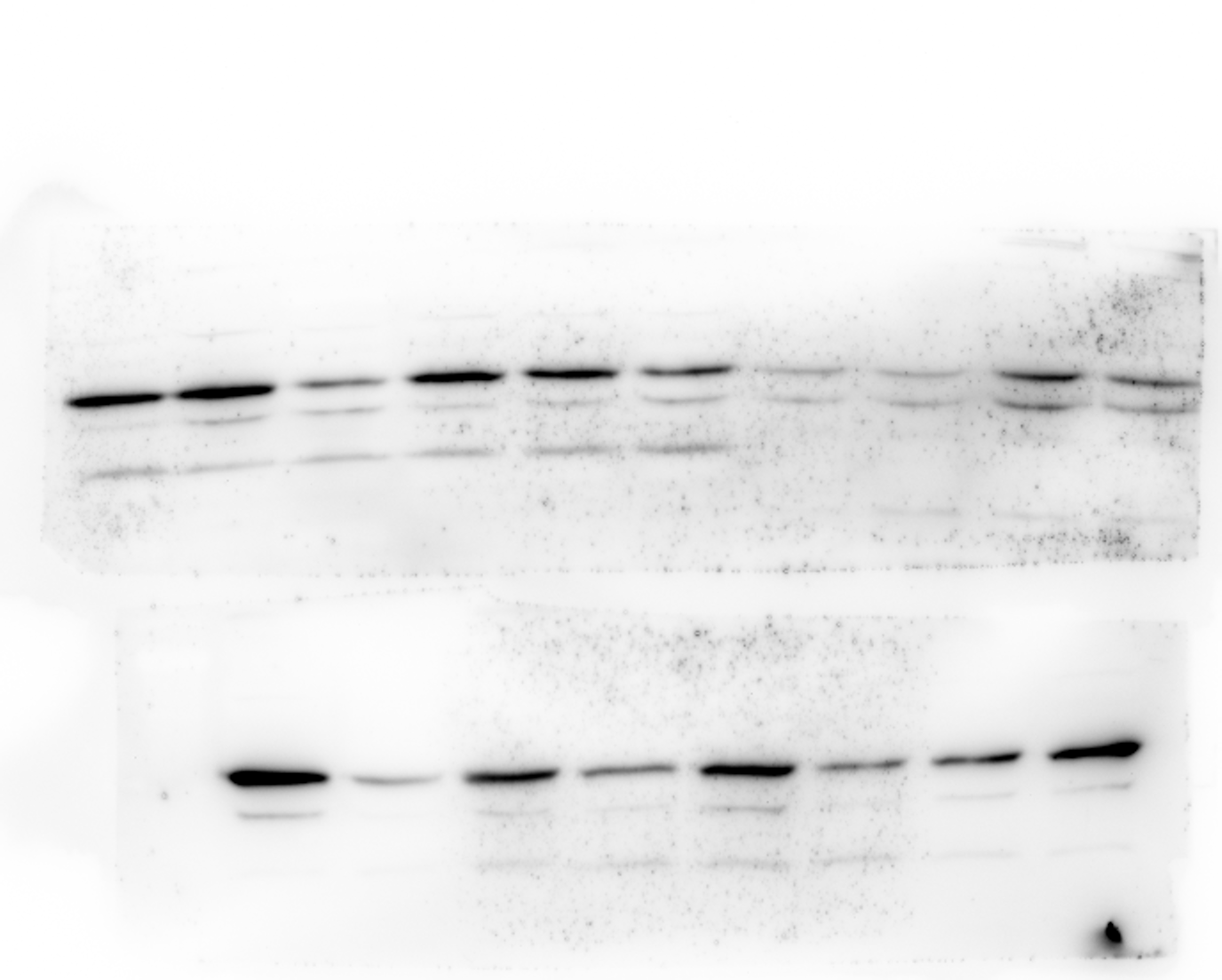

Supplement: Supplementary file 2 [file DataSheet_2.zip › Source data/Western Blot gel scan image/Fig. 4B/Fig. 4B-right—anti-Myc.Tif]

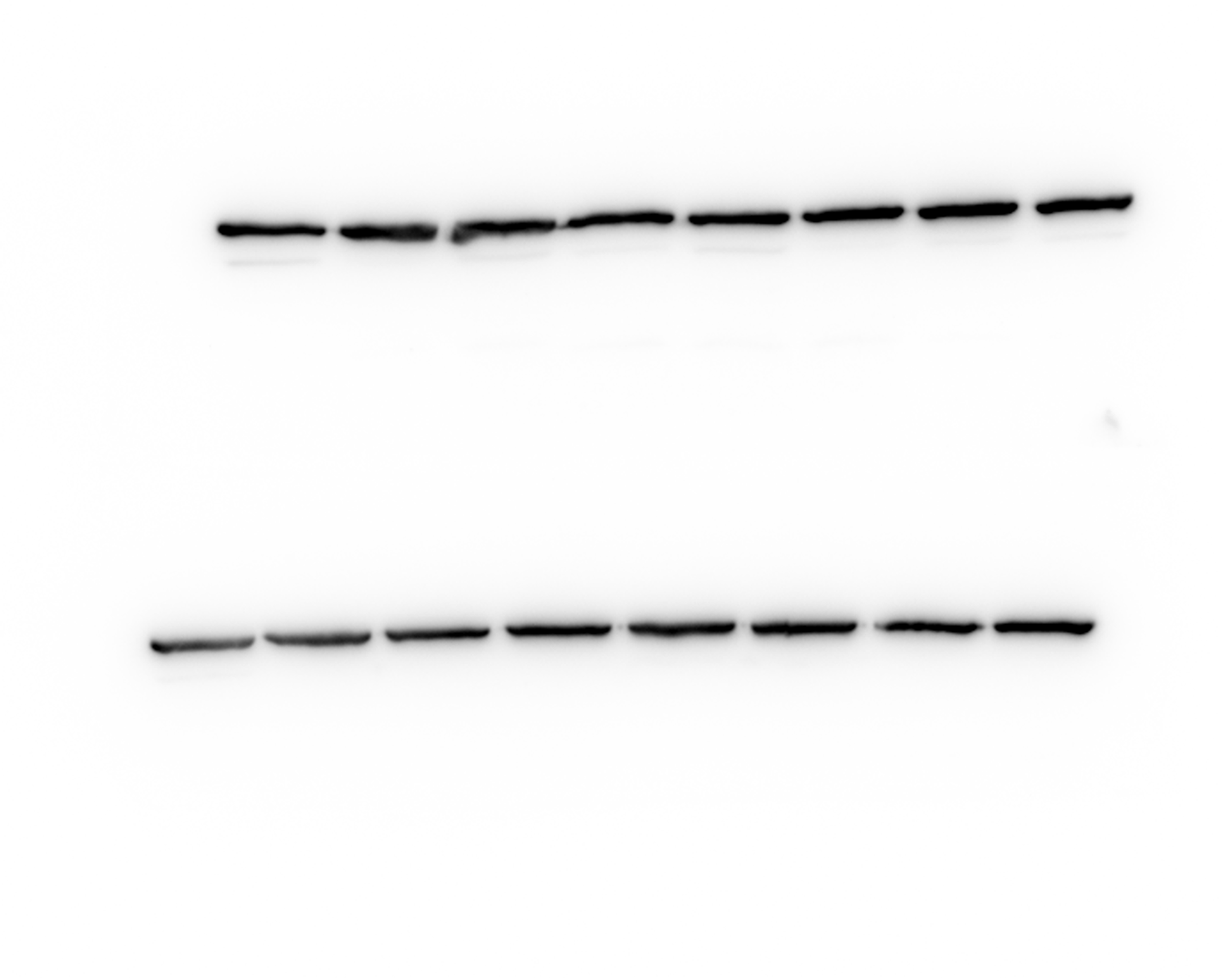

Supplement: Supplementary file 2 [file DataSheet_2.zip › Source data/Western Blot gel scan image/Fig. 4B/Fig. 4B-right—anti-Tubulin.Tif]

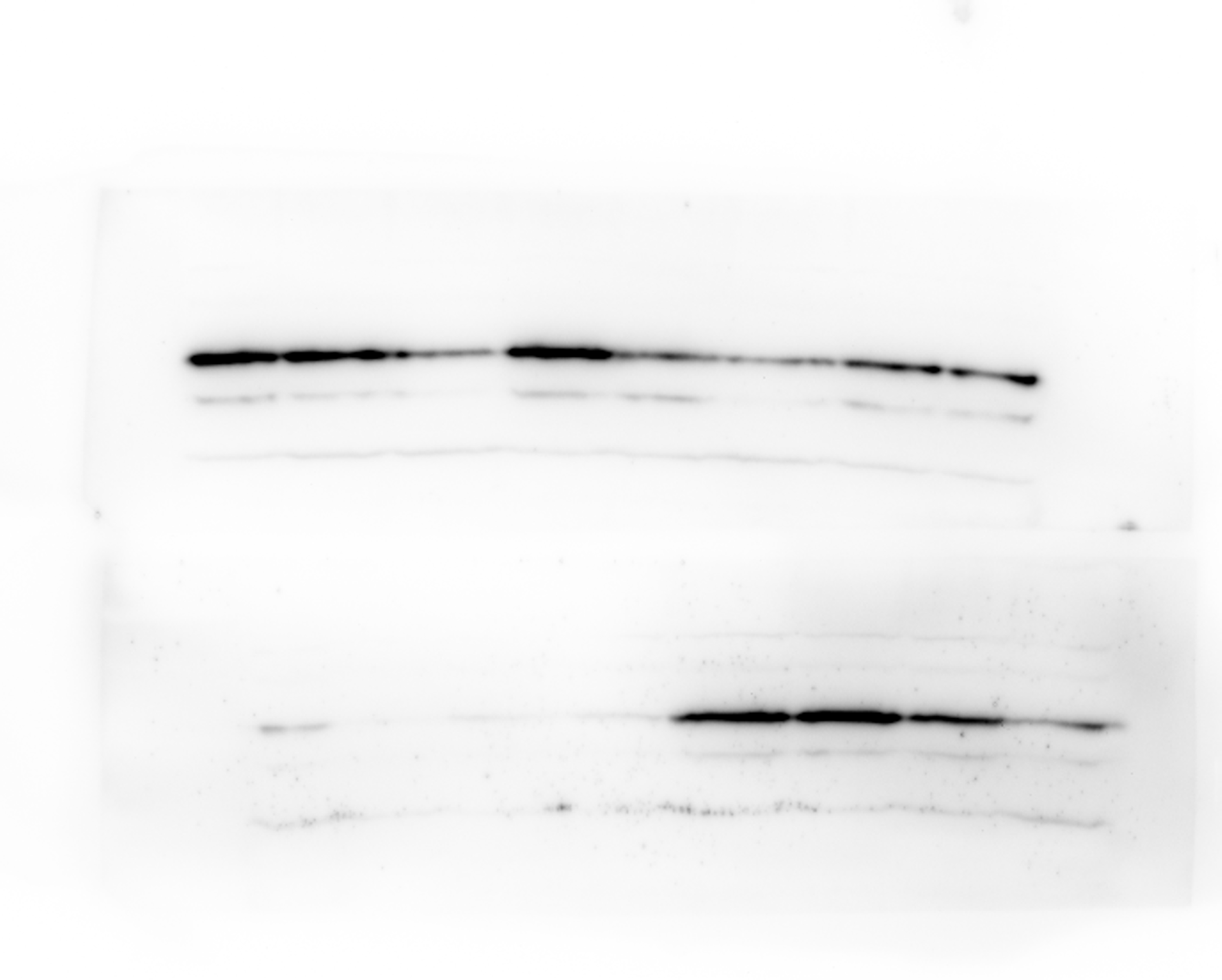

Supplement: Supplementary file 2 [file DataSheet_2.zip › Source data/Western Blot gel scan image/Fig. 4C/Fig. 4C-bottom—anti-Myc.Tif]

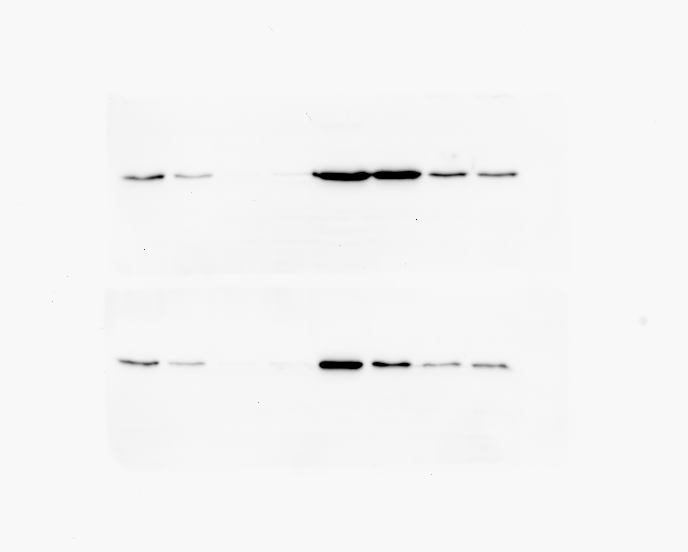

Supplement: Supplementary file 2 [file DataSheet_2.zip › Source data/Western Blot gel scan image/Fig. 4C/Fig. 4C-top—anti-Myc.tif]

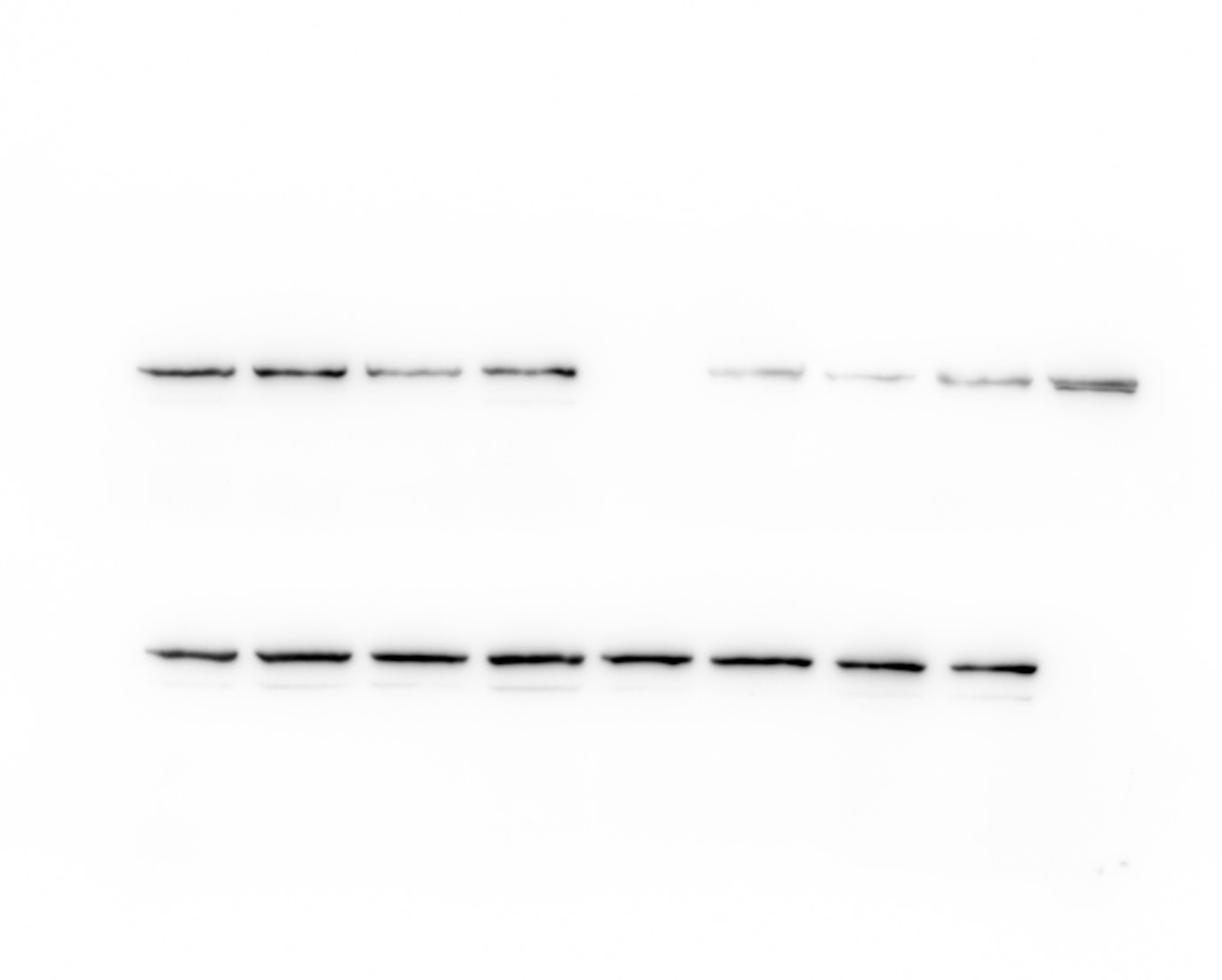

Supplement: Supplementary file 2 [file DataSheet_2.zip › Source data/Western Blot gel scan image/Fig. 4C/Fig. 4C-top—anti-Tubulin.Tif]

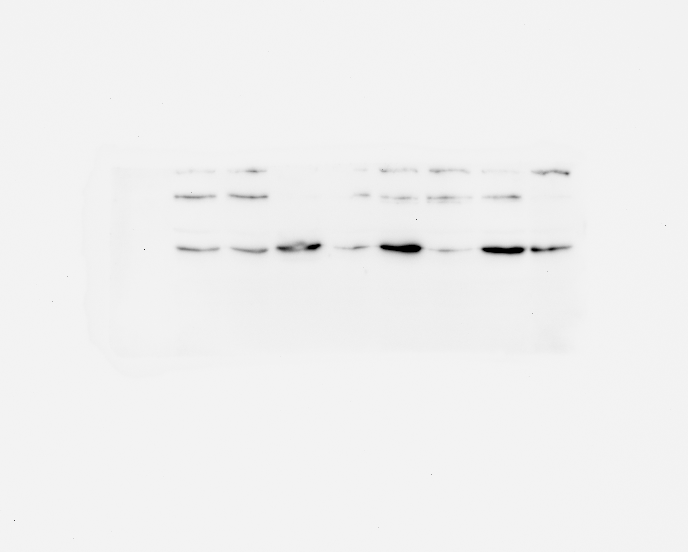

Supplement: Supplementary file 2 [file DataSheet_2.zip › Source data/Western Blot gel scan image/Fig. 5B/Fig. 5B-top—anti-Myc.tif]

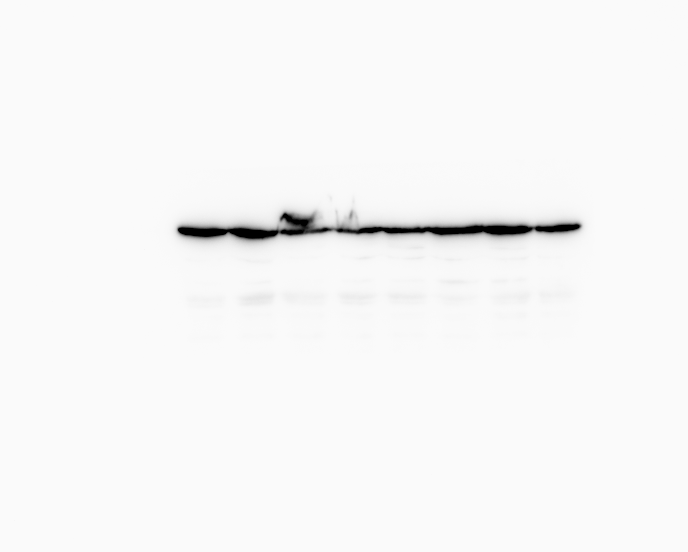

Supplement: Supplementary file 2 [file DataSheet_2.zip › Source data/Western Blot gel scan image/Fig. 5B/Fig. 5B-top—anti-Tubulin.tif]

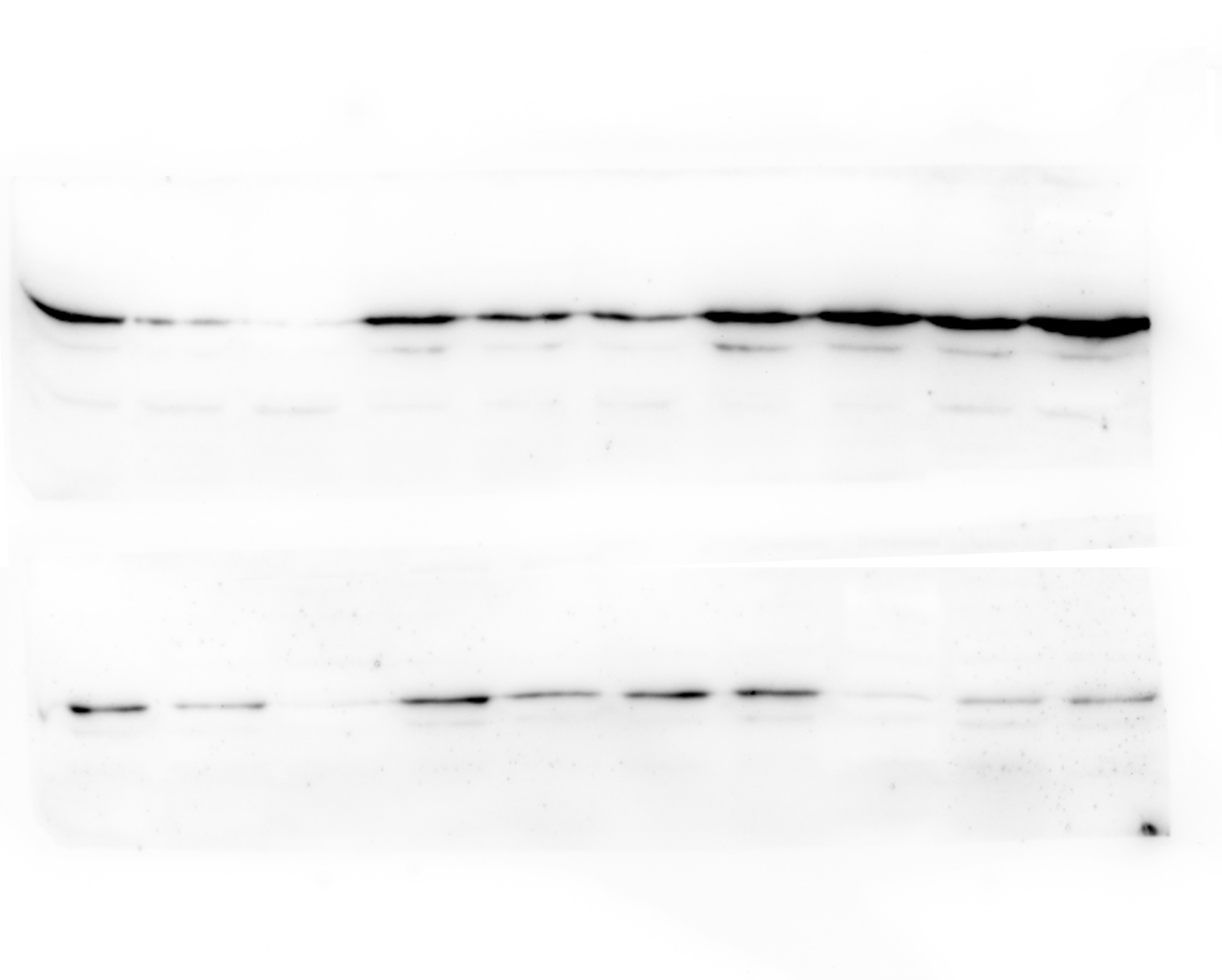

Supplement: Supplementary file 2 [file DataSheet_2.zip › Source data/Western Blot gel scan image/Fig. 5C/Fig. 5C-bottom—anti-Myc.Tif]

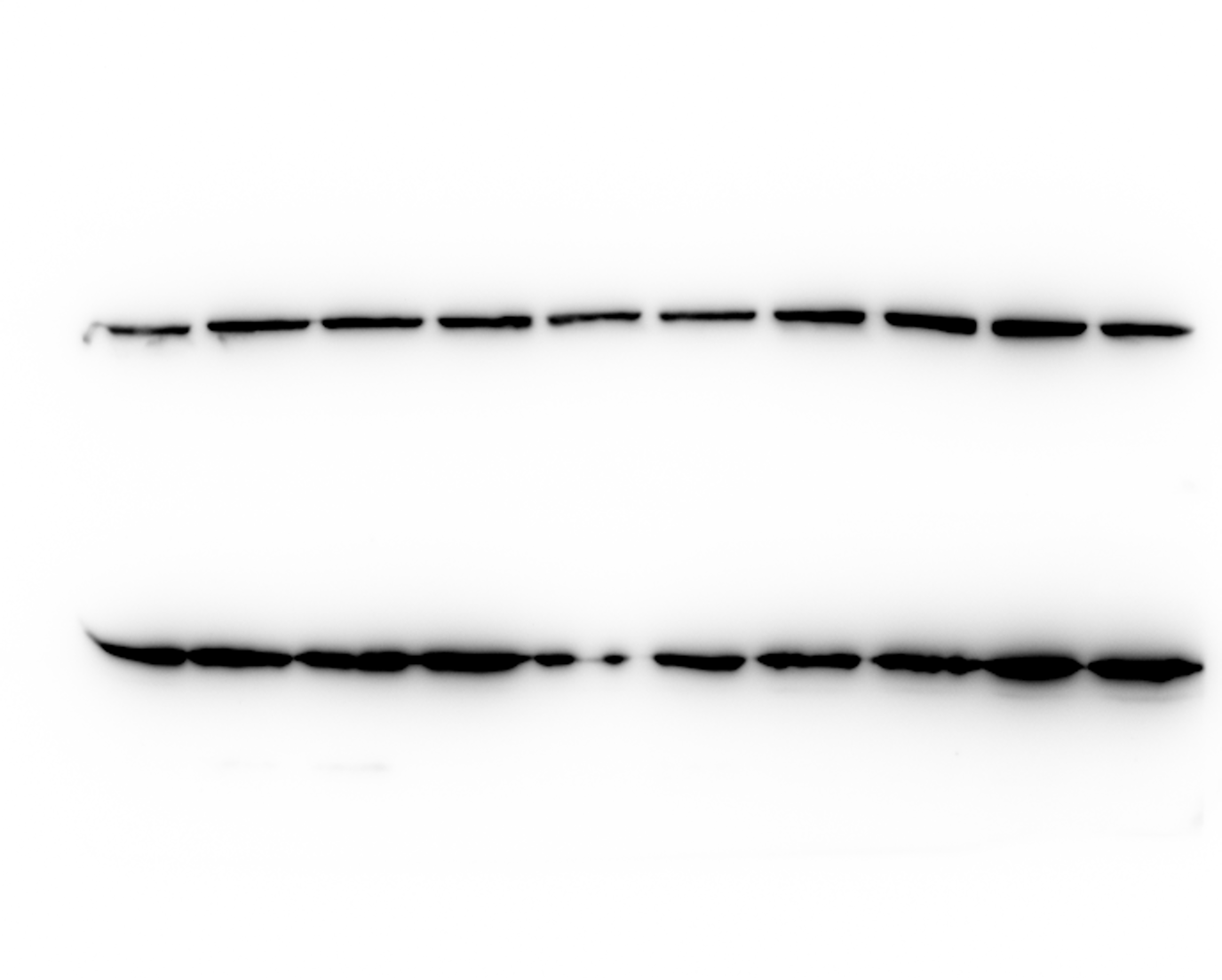

Supplement: Supplementary file 2 [file DataSheet_2.zip › Source data/Western Blot gel scan image/Fig. 5C/Fig. 5C-bottom—anti-Tubulin.Tif]

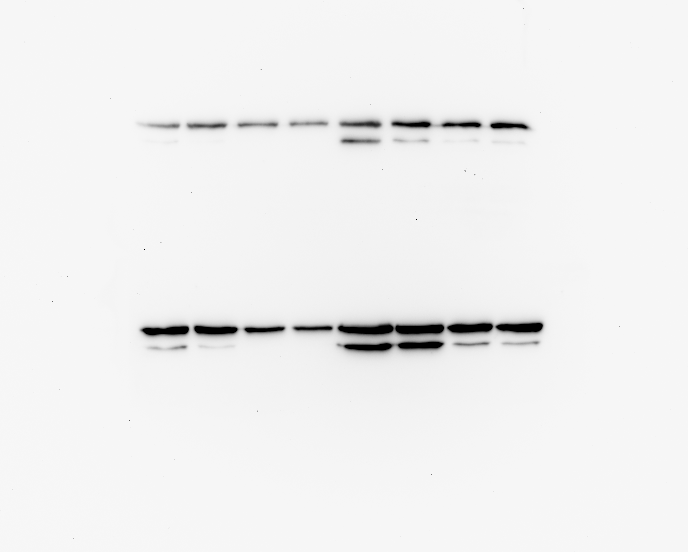

Supplement: Supplementary file 2 [file DataSheet_2.zip › Source data/Western Blot gel scan image/Fig. 5C/Fig. 5C-top—anti-Myc+Tubulin.tif]

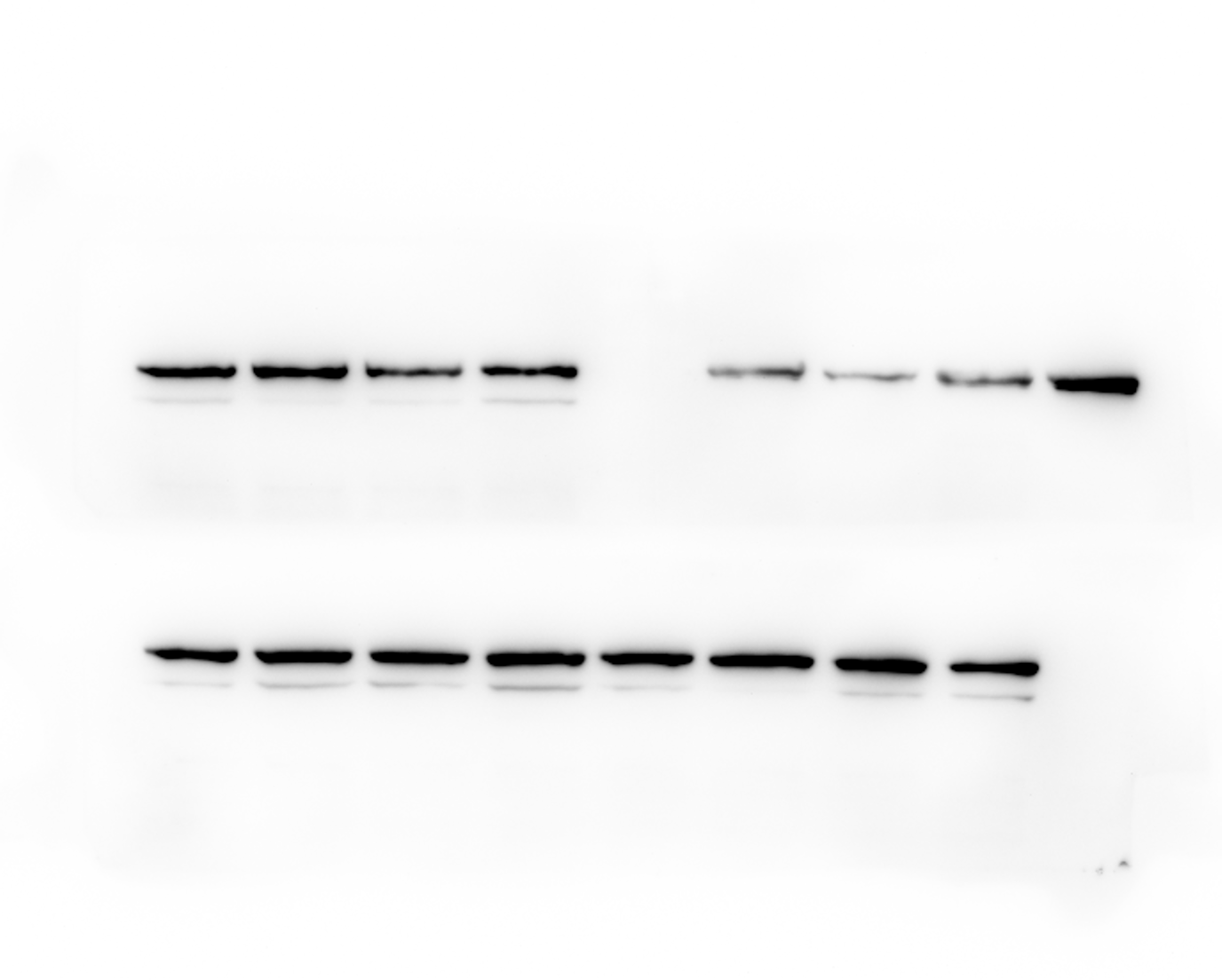

Supplement: Supplementary file 2 [file DataSheet_2.zip › Source data/Western Blot gel scan image/Fig. 6A/Fig. 6A-left—anti-Myc.Tif]

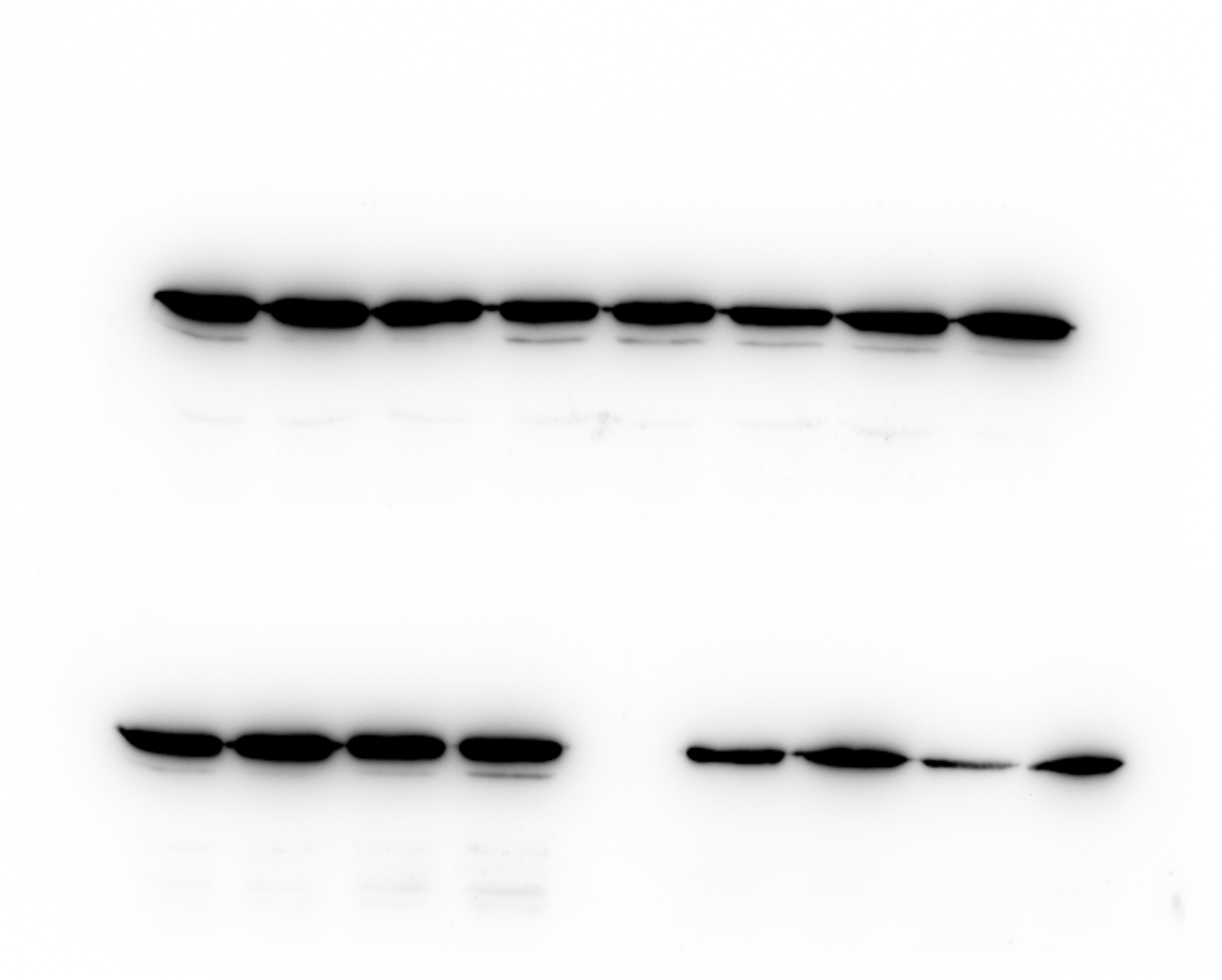

Supplement: Supplementary file 2 [file DataSheet_2.zip › Source data/Western Blot gel scan image/Fig. 6A/Fig. 6A-left—anti-Tubulin.Tif]

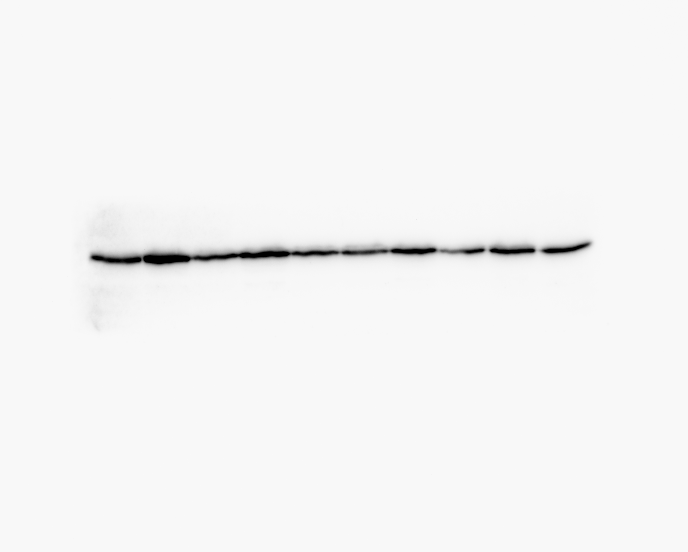

Supplement: Supplementary file 2 [file DataSheet_2.zip › Source data/Western Blot gel scan image/Fig. 6A/Fig. 6A-right—anti-Myc.tif]

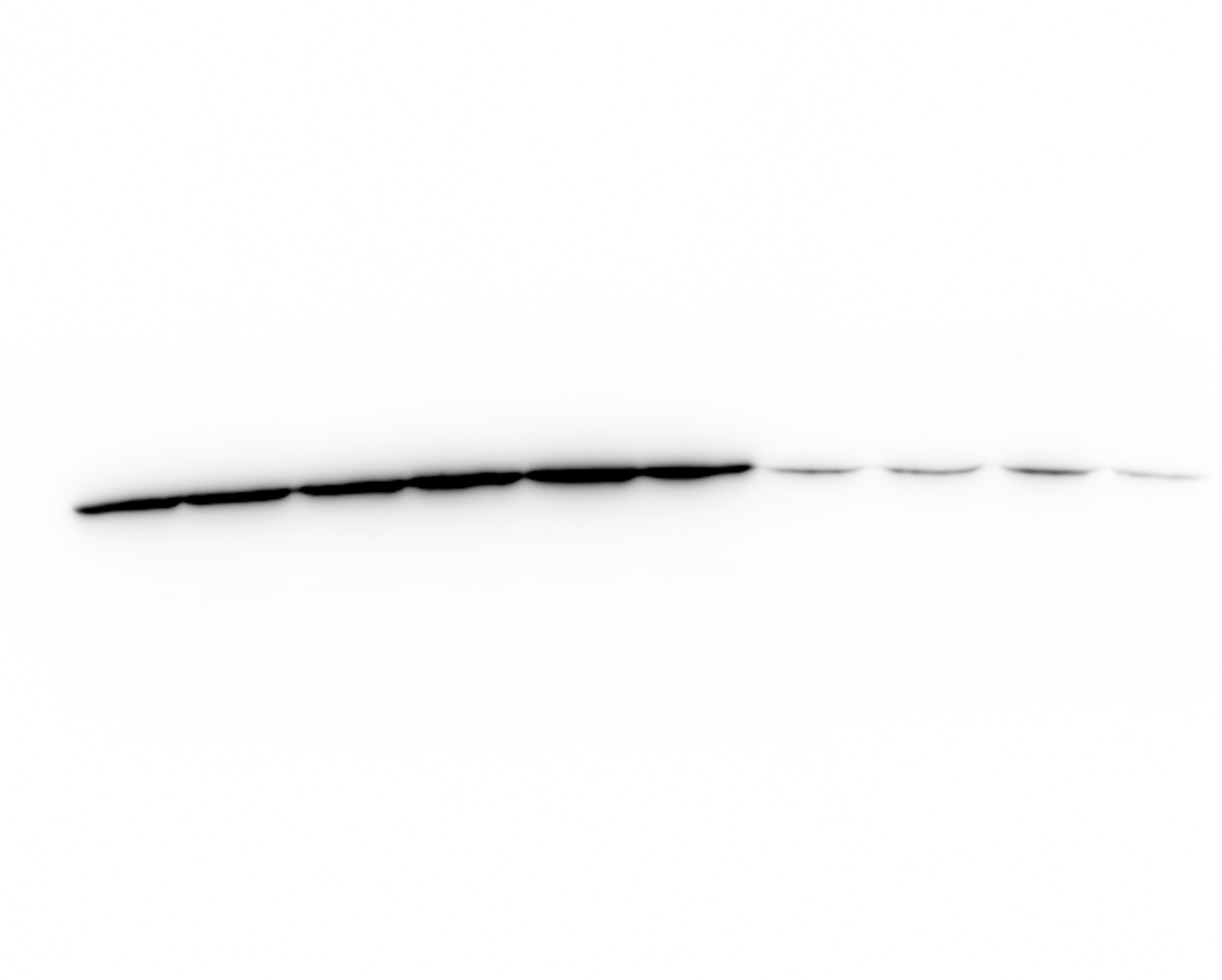

Supplement: Supplementary file 2 [file DataSheet_2.zip › Source data/Western Blot gel scan image/Fig. 6A/Fig. 6A-right—anti-Tubulin.Tif]

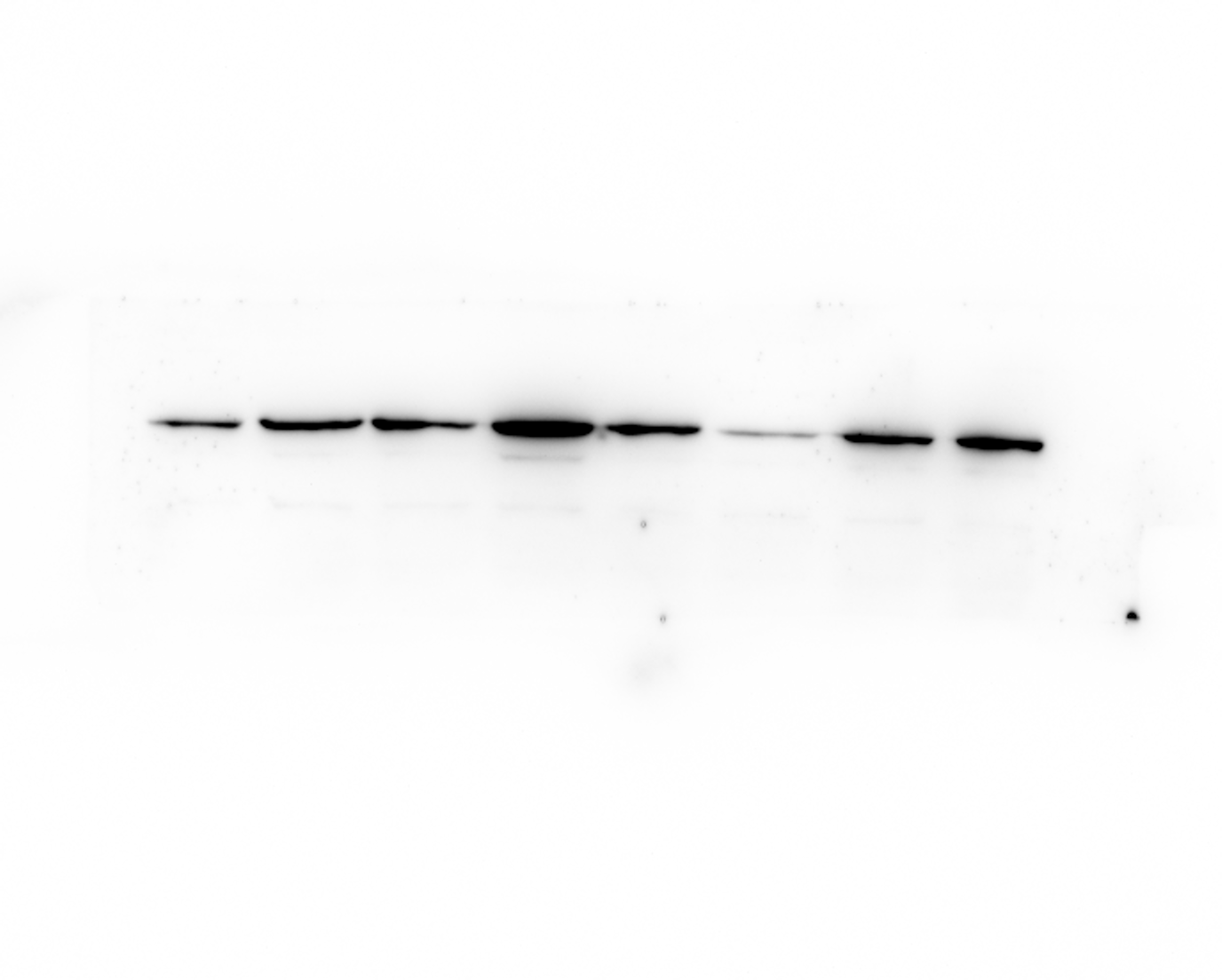

Supplement: Supplementary file 2 [file DataSheet_2.zip › Source data/Western Blot gel scan image/Fig. 6B/Fig. 6B-left—anti-Myc.Tif]

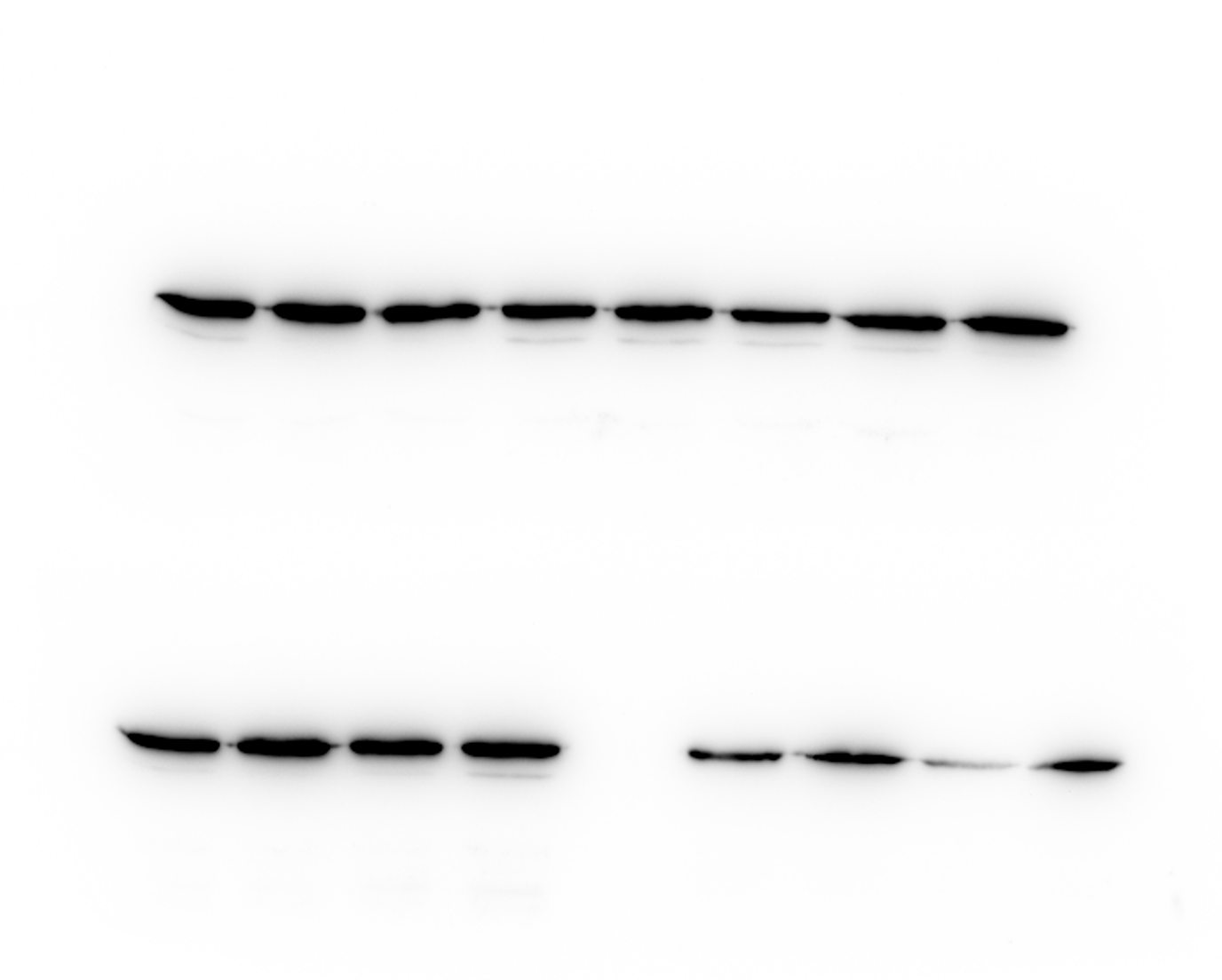

Supplement: Supplementary file 2 [file DataSheet_2.zip › Source data/Western Blot gel scan image/Fig. 6B/Fig. 6B-left—anti-Tubulin.Tif]

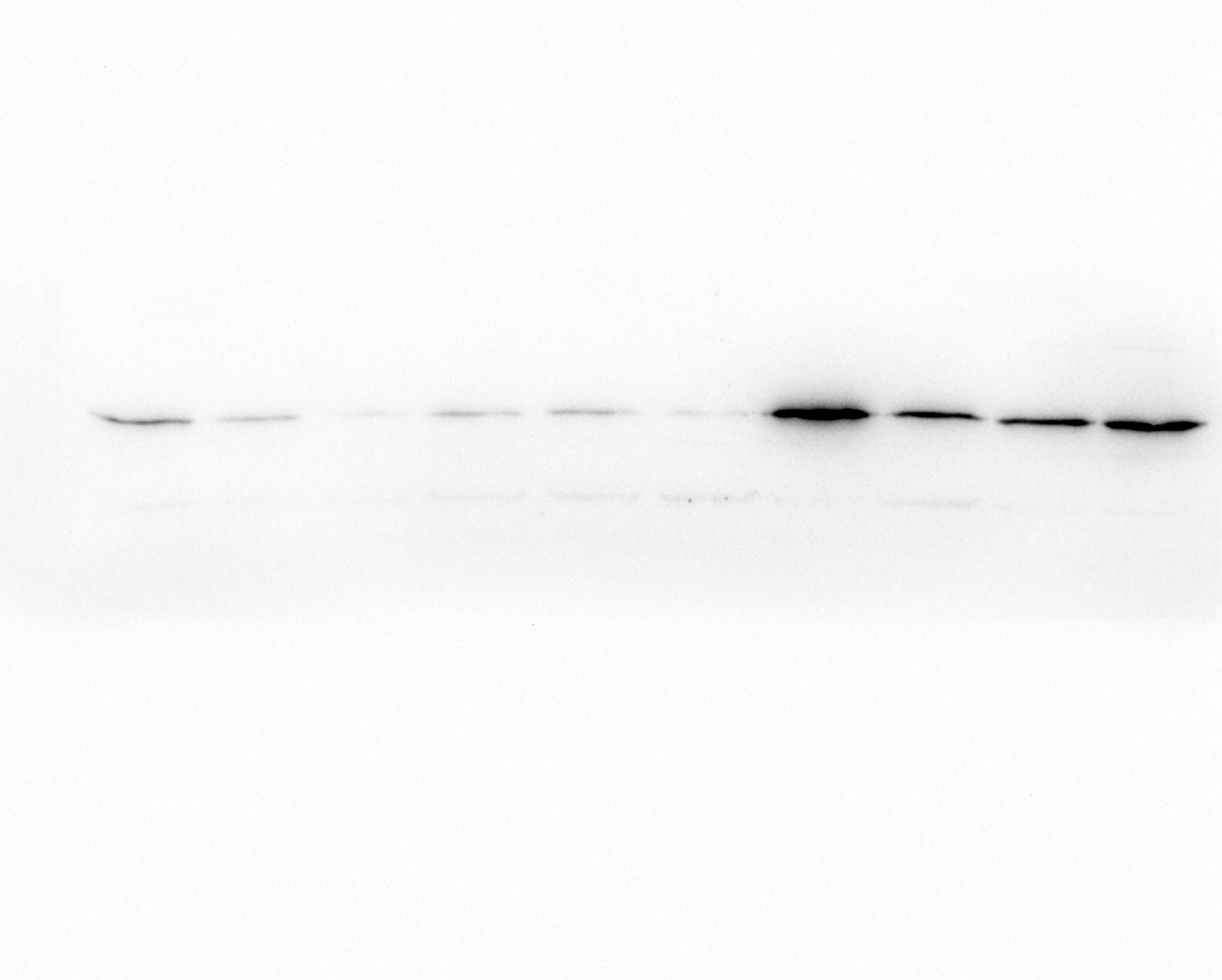

Supplement: Supplementary file 2 [file DataSheet_2.zip › Source data/Western Blot gel scan image/Fig. 6B/Fig. 6B-right—anti-Myc.Tif]

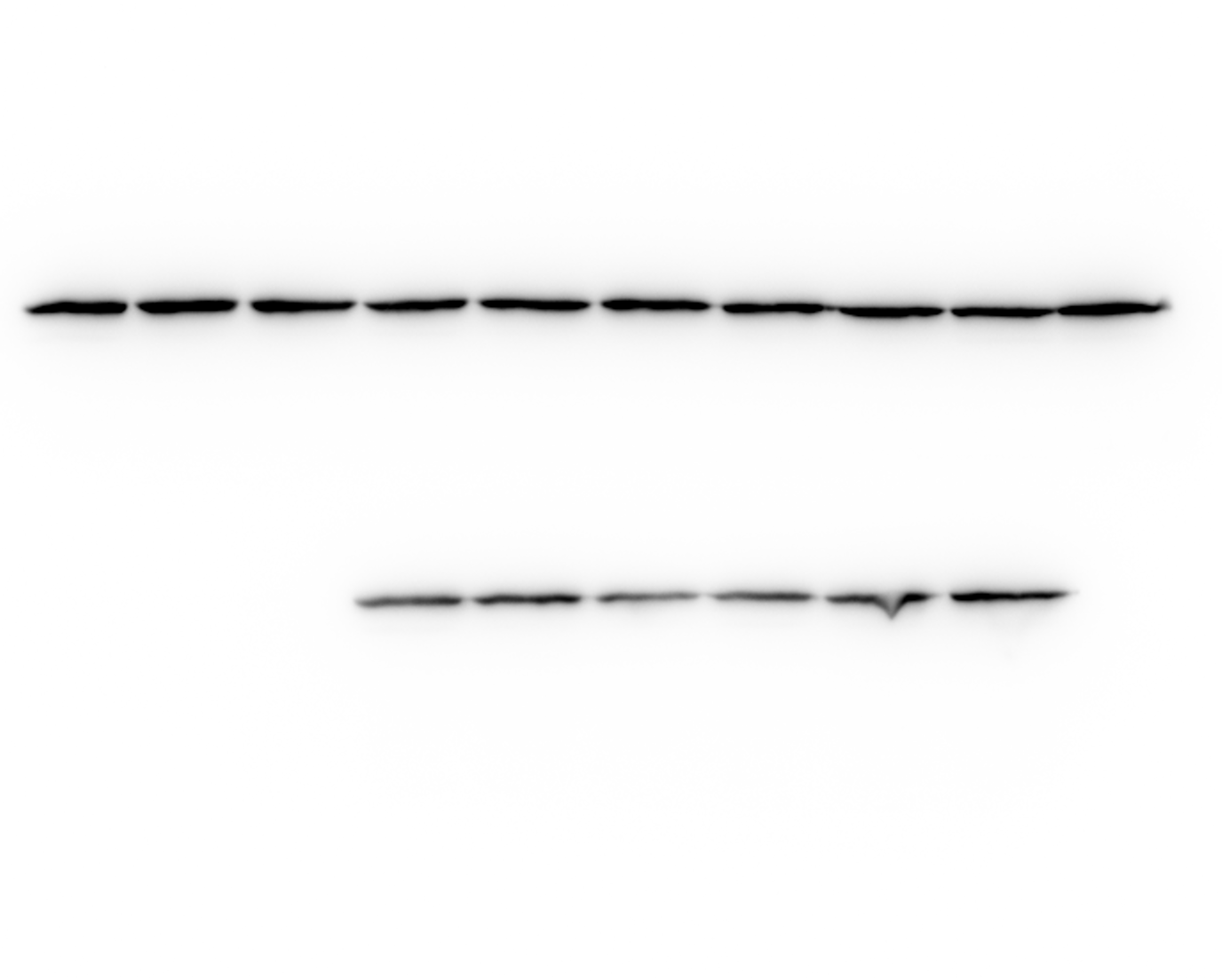

Supplement: Supplementary file 2 [file DataSheet_2.zip › Source data/Western Blot gel scan image/Fig. 6B/Fig. 6B-right—anti-Tubulin.Tif]

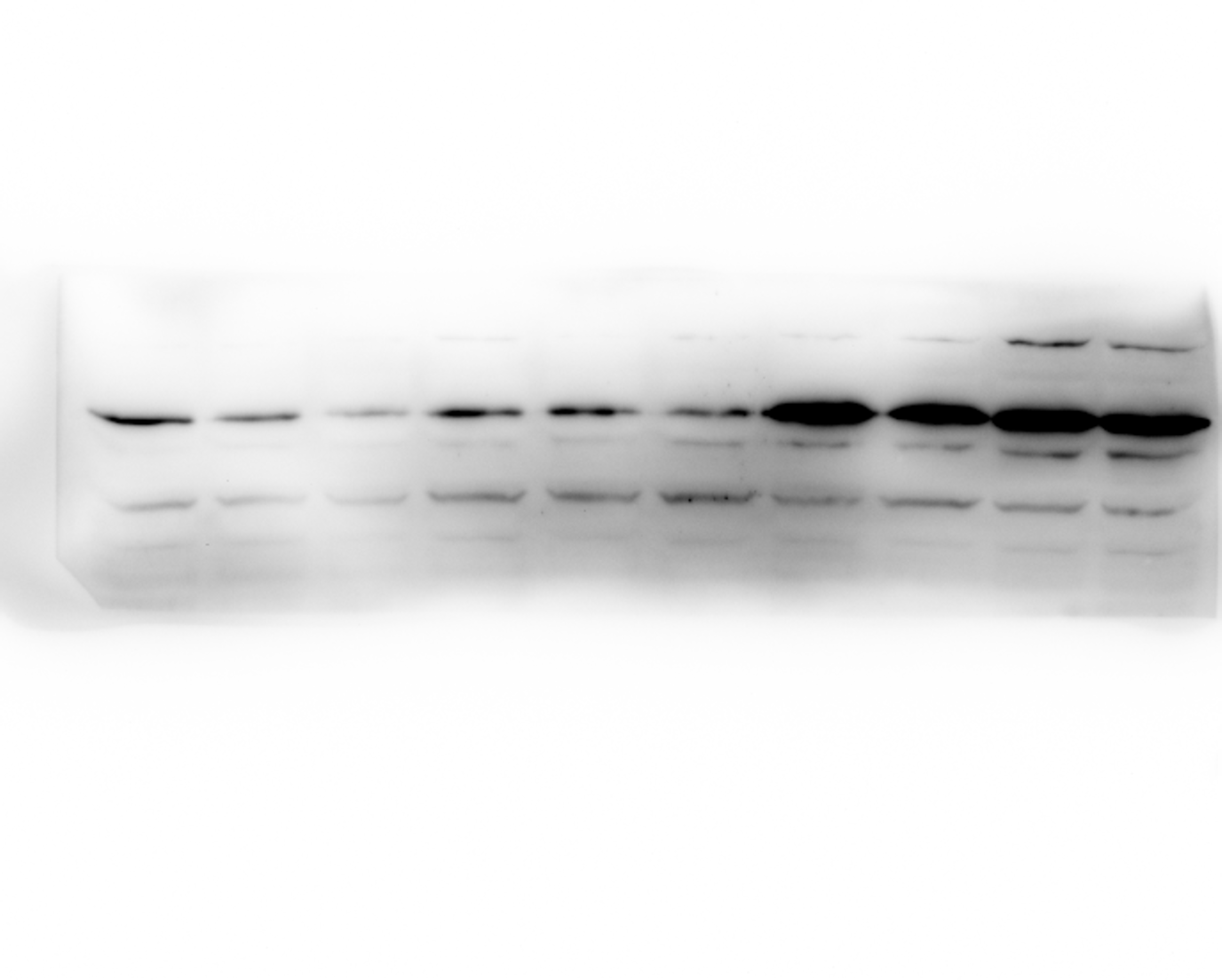

Supplement: Supplementary file 2 [file DataSheet_2.zip › Source data/Western Blot gel scan image/Fig. 6C/Fig. 6C-bottom—anti-Myc.Tif]

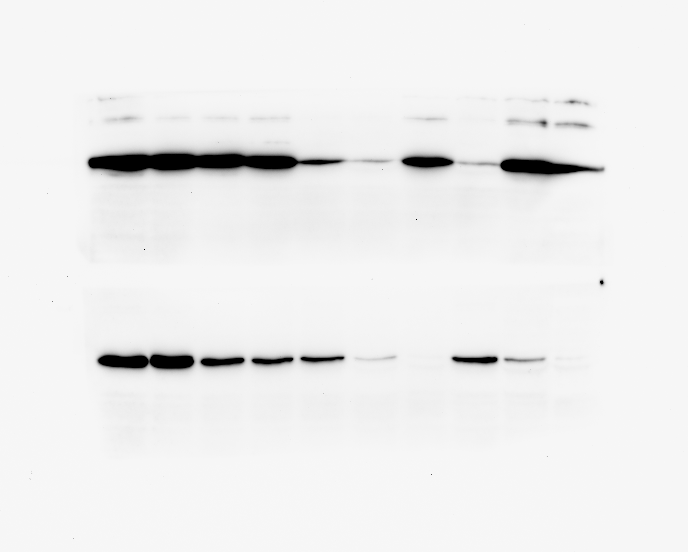

Supplement: Supplementary file 2 [file DataSheet_2.zip › Source data/Western Blot gel scan image/Fig. 6C/Fig. 6C-top—anti-Myc.tif]

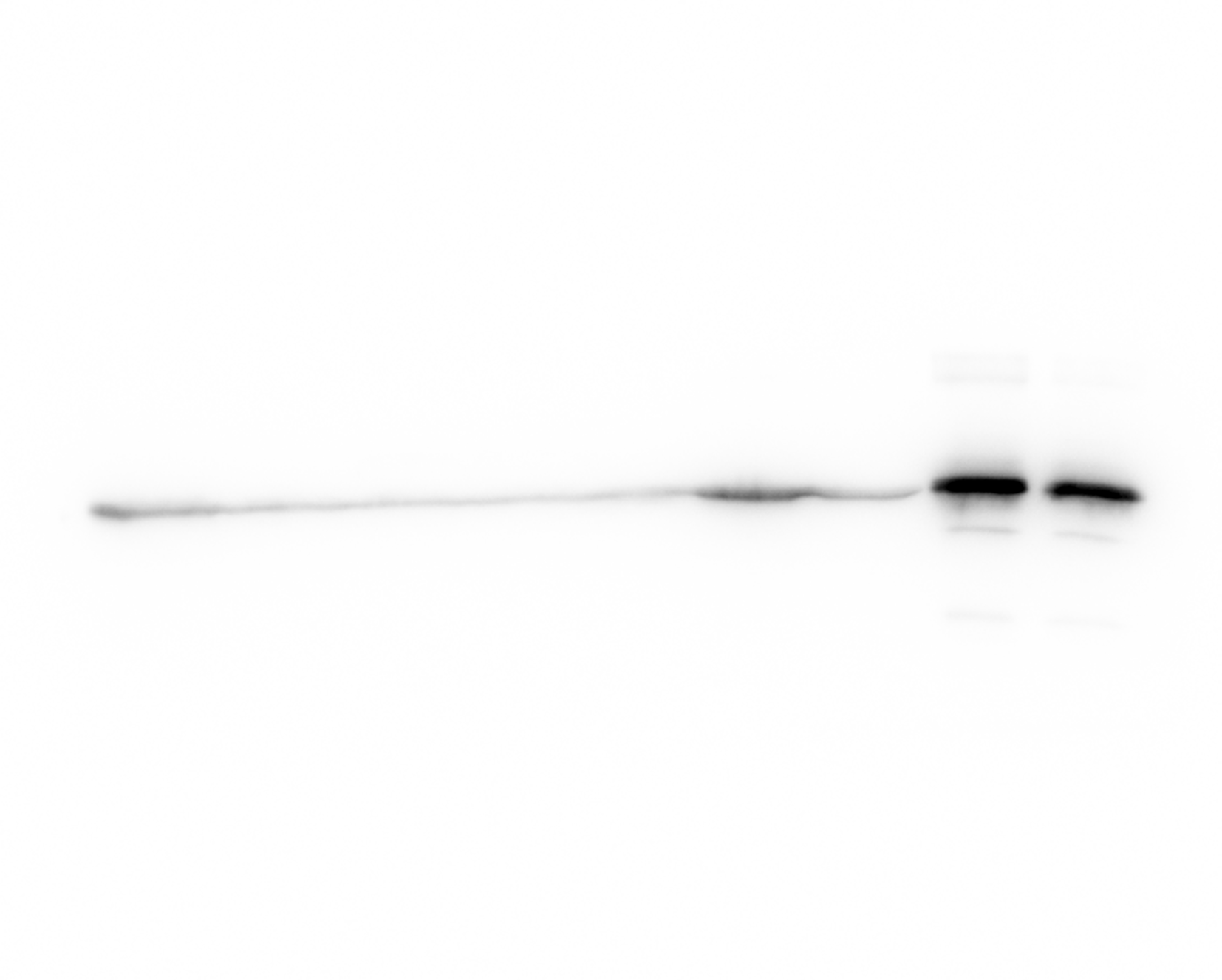

Supplement: Supplementary file 2 [file DataSheet_2.zip › Source data/Western Blot gel scan image/Fig. 7A/Fig. 7A-left-IP—anti-Myc.Tif]

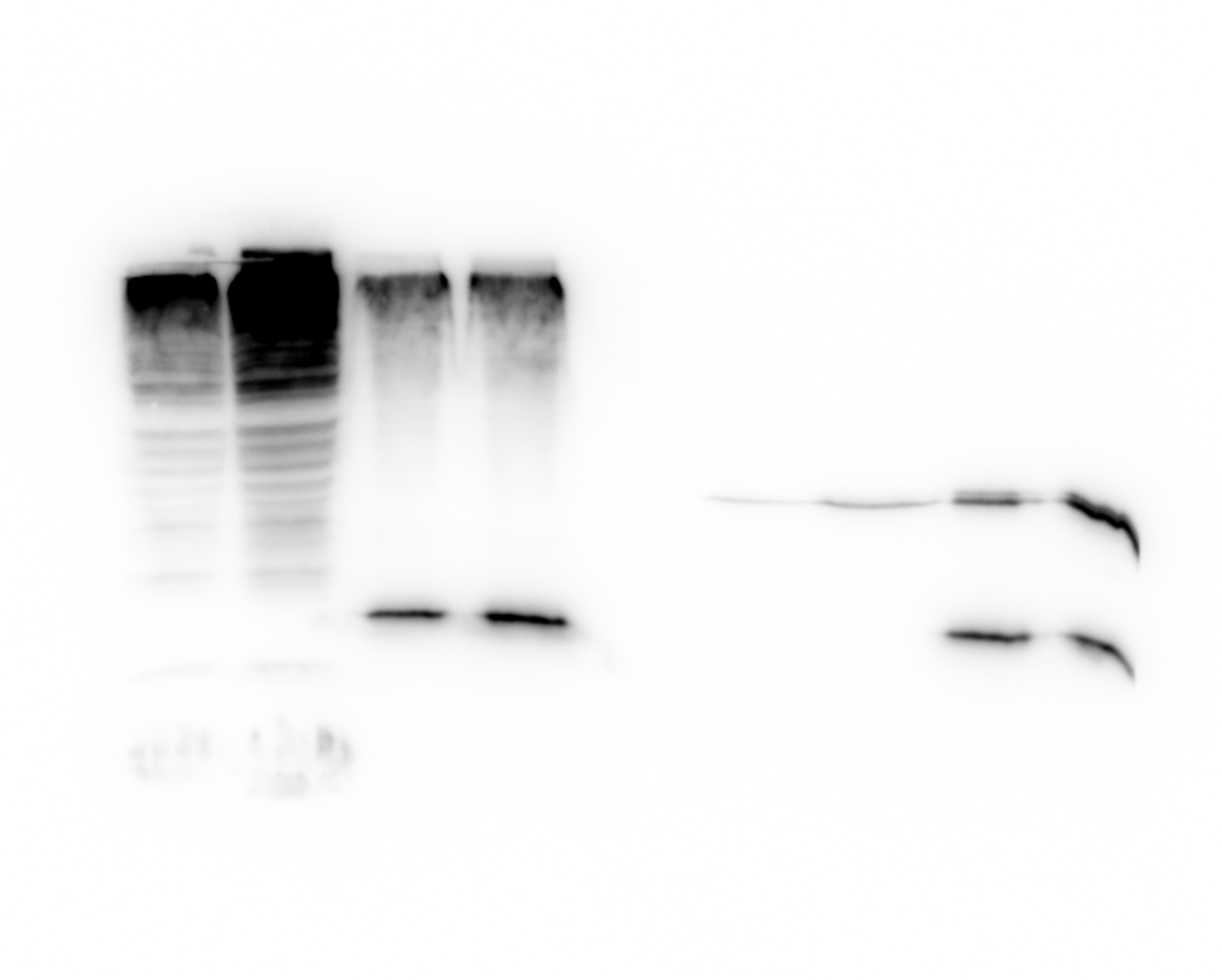

Supplement: Supplementary file 2 [file DataSheet_2.zip › Source data/Western Blot gel scan image/Fig. 7A/Fig. 7A-left-WCL+IP—anti-HA.Tif]

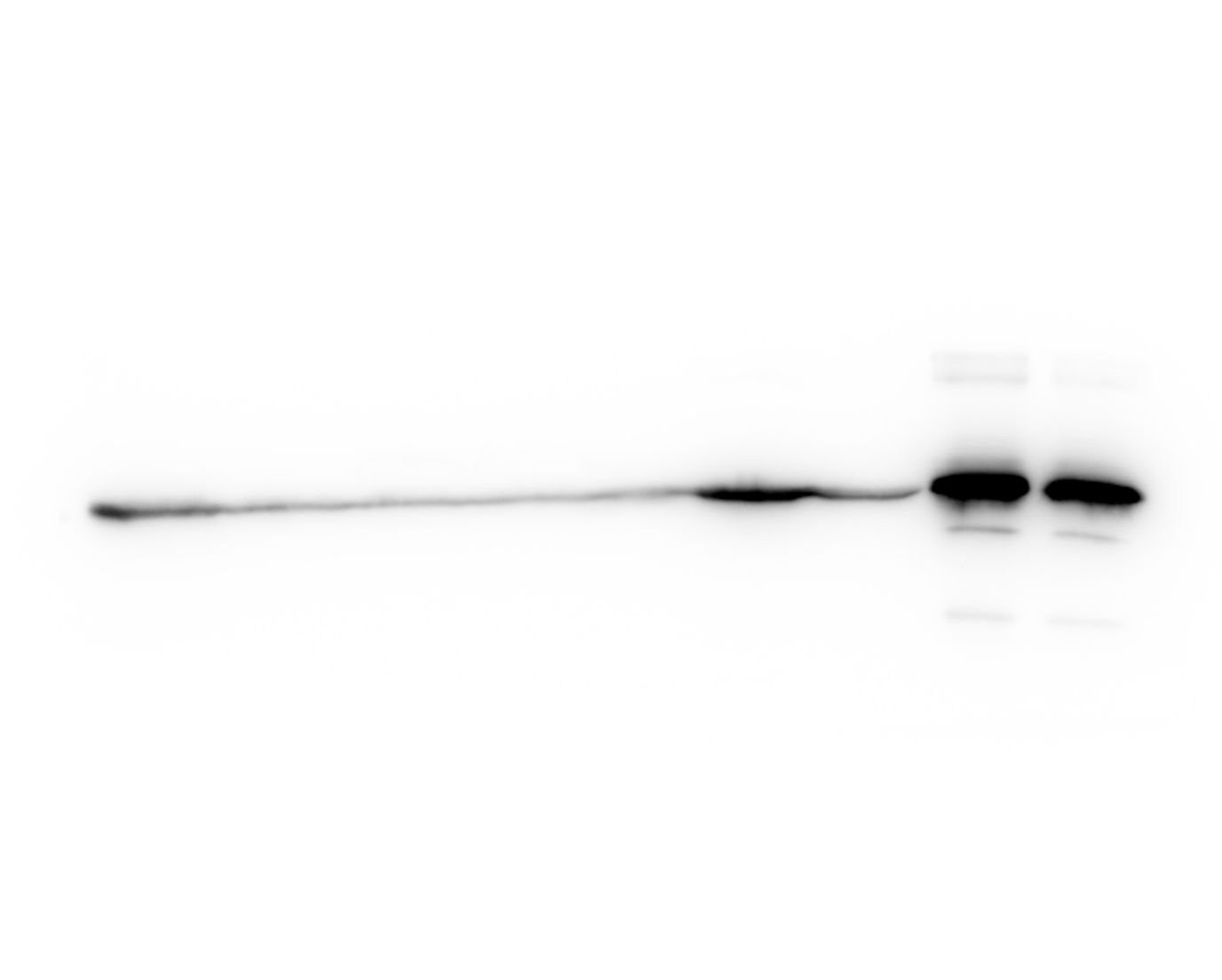

Supplement: Supplementary file 2 [file DataSheet_2.zip › Source data/Western Blot gel scan image/Fig. 7A/Fig. 7A-left-WCL—anti-Myc.Tif]

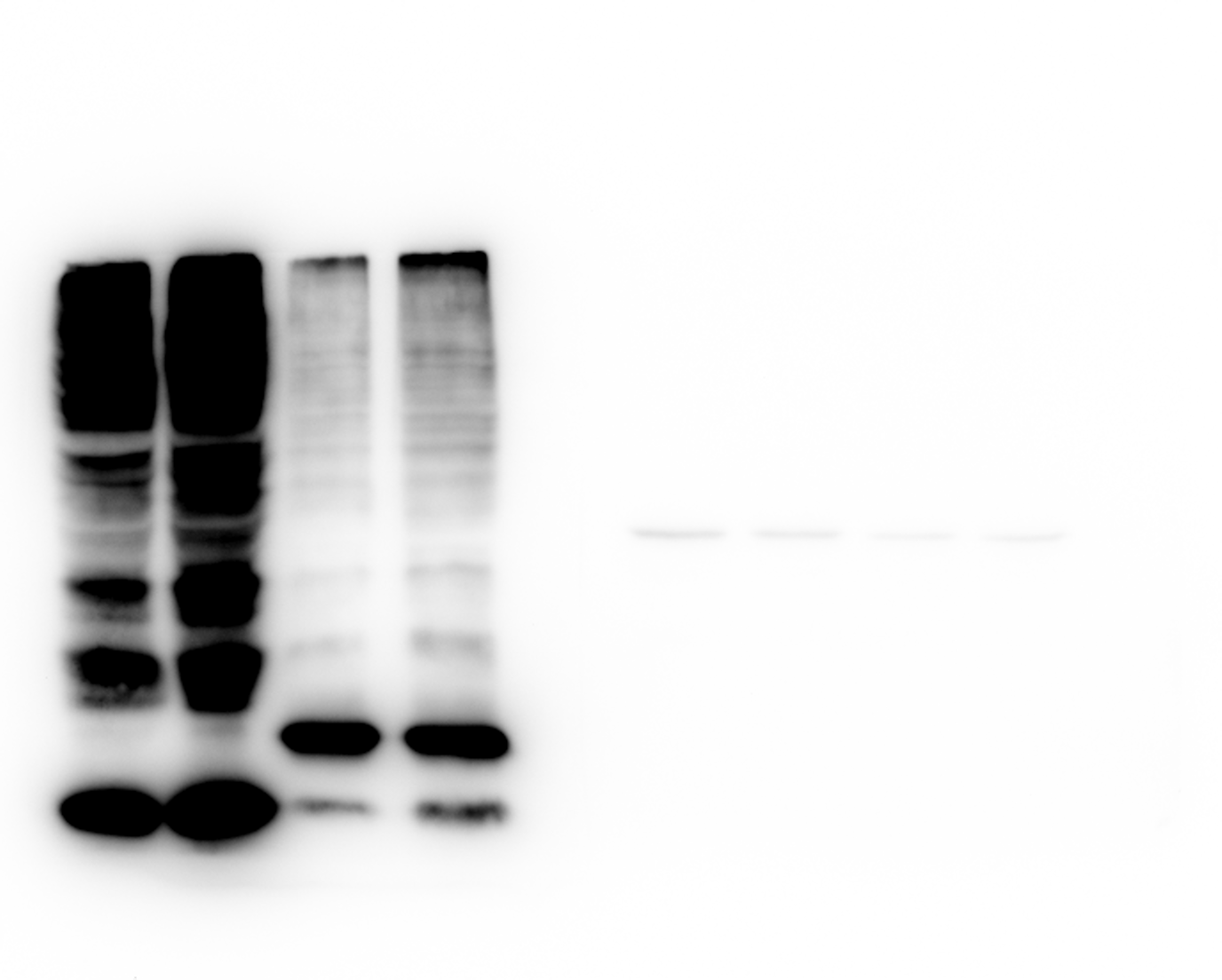

Supplement: Supplementary file 2 [file DataSheet_2.zip › Source data/Western Blot gel scan image/Fig. 7A/Fig. 7A-right-WCL+IP—anti-HA.Tif]

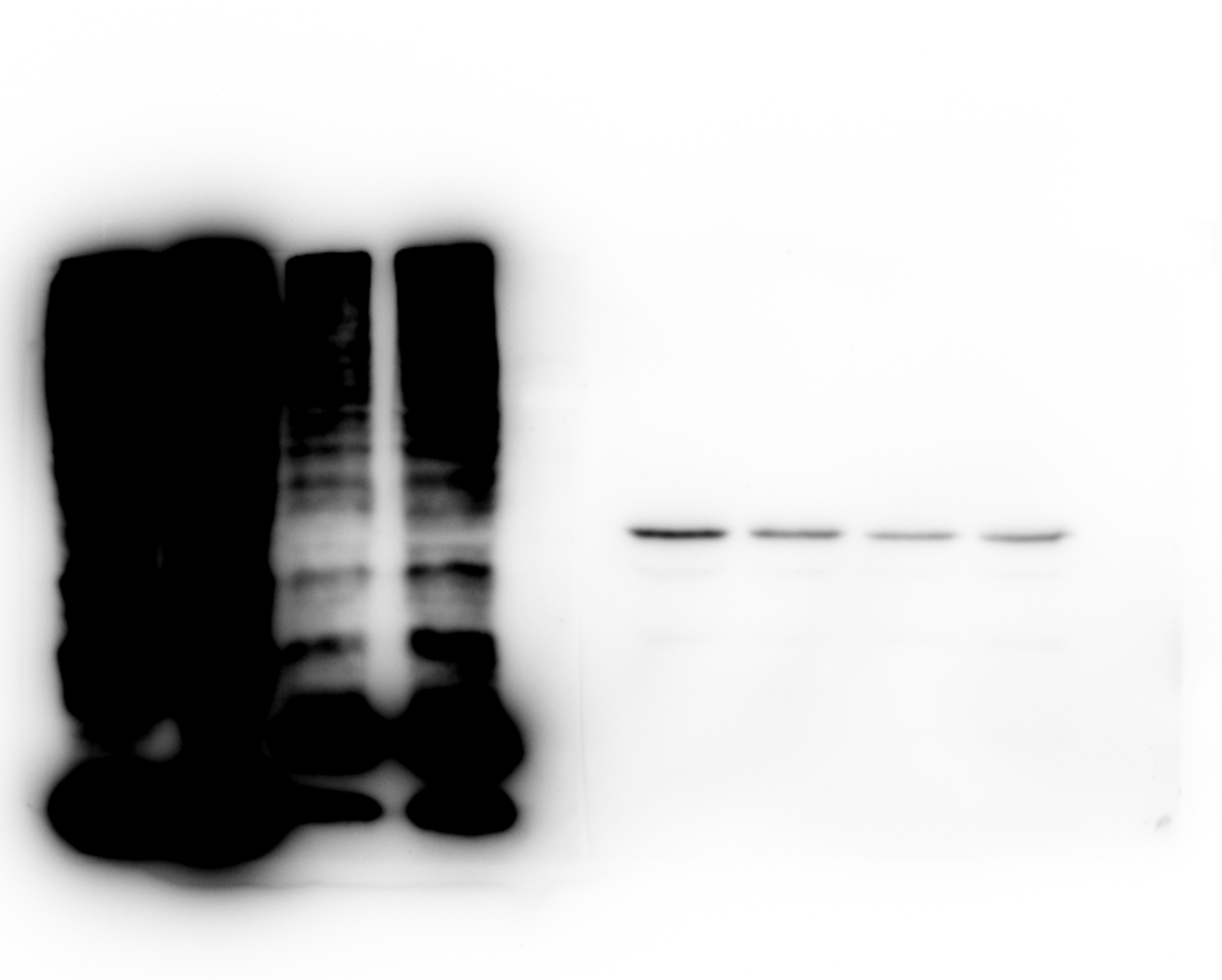

Supplement: Supplementary file 2 [file DataSheet_2.zip › Source data/Western Blot gel scan image/Fig. 7A/Fig. 7A-right-WCL+IP—anti-Myc.Tif]

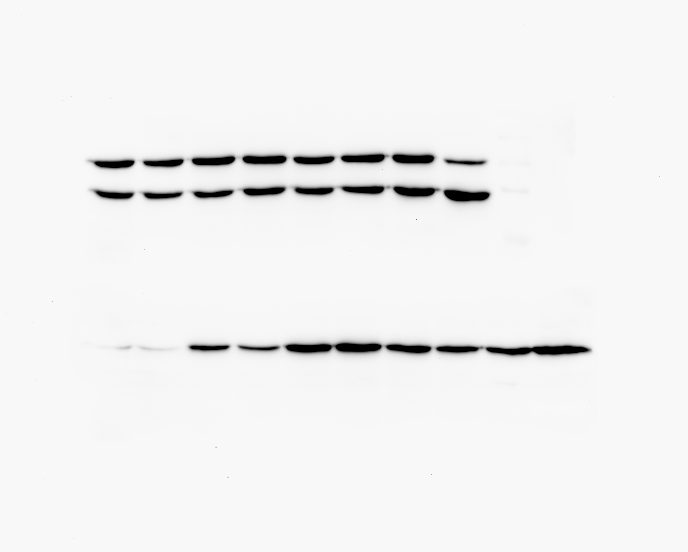

Supplement: Supplementary file 2 [file DataSheet_2.zip › Source data/Western Blot gel scan image/Fig. 7B/Fig. 7B-bottom—anti-GAPDH.tif]

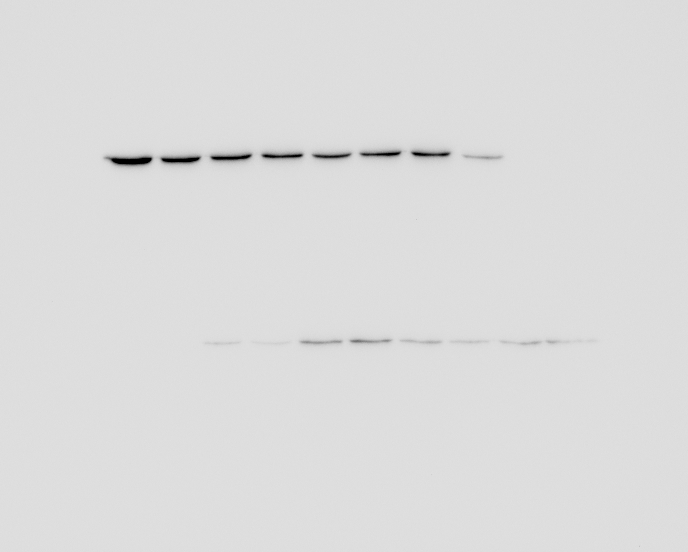

Supplement: Supplementary file 2 [file DataSheet_2.zip › Source data/Western Blot gel scan image/Fig. 7B/Fig. 7B-bottom—anti-Myc.tif]

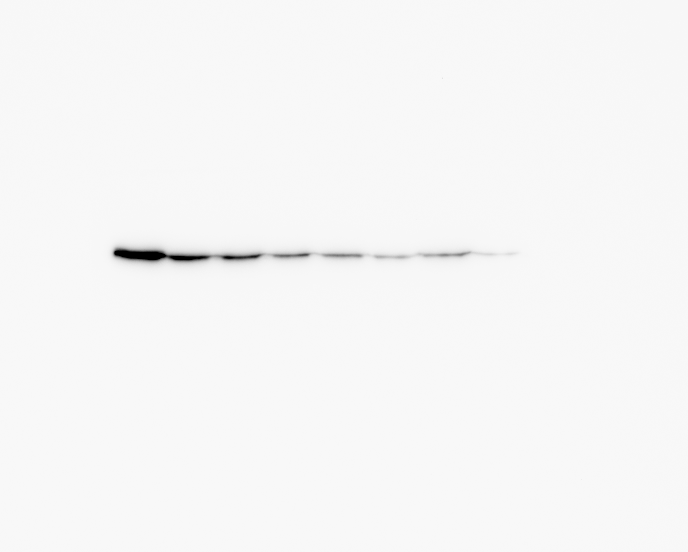

Supplement: Supplementary file 2 [file DataSheet_2.zip › Source data/Western Blot gel scan image/Fig. 7B/Fig. 7B-top—anti-Myc.tif]

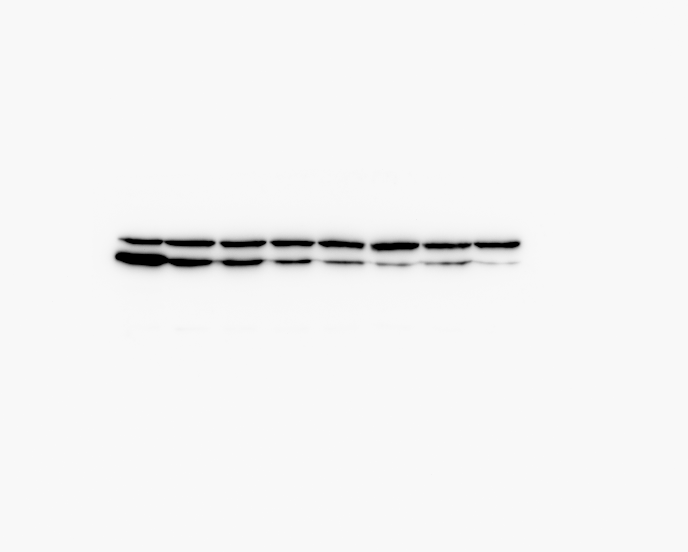

Supplement: Supplementary file 2 [file DataSheet_2.zip › Source data/Western Blot gel scan image/Fig. 7B/Fig. 7B-top—anti-Tubulin.tif]
